# Global Implications of Local Unfolding Phenomena, Probed by Cysteine Reactivity in Human Frataxin

**DOI:** 10.1038/s41598-019-39429-2

**Published:** 2019-02-11

**Authors:** Santiago E. Faraj, Martín E. Noguera, José María Delfino, Javier Santos

**Affiliations:** 1 Alejandro Paladini Institute of Biological Chemistry and Chemical Physics (UBA-CONICET), Faculty of Pharmacy and Biochemistry, University of Buenos Aires, Junín 956, (C1113AAD), Buenos Aires, Argentina; 2 Departamento de Fisiología y Biología Molecular y Celular, Facultad de Ciencia Exactas y Naturales, Universidad de Buenos Aires. Instituto de Biociencias, Biotecnología y Biomedicina (iB3). Intendente Güiraldes 2160 - Ciudad Universitaria, 1428EGA C.A.B.A., Argentina

**Keywords:** Biophysical chemistry, Biophysics

## Abstract

Local events that affect specific regions of proteins are of utmost relevance for stability and function. The aim of this study is to quantitatively assess the importance of locally-focused dynamics by means of a simple chemical modification procedure. Taking human Frataxin as a working model, we investigated local fluctuations of the C-terminal region (the last 16 residues of the protein) by means of three L → C replacement mutants: L98C, L200C and L203C. The conformation and thermodynamic stability of each variant was assessed. All the variants exhibited native features and high stabilities: 9.1 (wild type), 8.1 (L198C), 7.0 (L200C) and 10.0 kcal mol^−1^ (L203C). In addition, kinetic rates of Cys chemical modification by DTNB and DTDPy were measured, conformational dynamics data were extracted and free energy for the local unfolding of the C-terminal region was estimated. The analysis of these results indicates that the conformation of the C-terminal region fluctuates with partial independence from global unfolding events. Additionally, numerical fittings of the kinetic model of the process suggest that the local transition occurs in the seconds to minutes timescale. In fact, standard free energy differences for local unfolding were found to be significantly lower than those of the global unfolding reaction, showing that chemical modification results may not be explained in terms of the global unfolding reaction alone. These results provide unequivocal experimental evidence of local phenomena with global effects and contribute to understanding how global and local stability are linked to protein dynamics.

## Introduction

Even though proteins tend to fold into compact conformations of roughly equal stability that minimize their energy and maximize the system’s entropy, they are not rigid, but flexible objects with dynamics that allow them to interact with the surroundings and accomplish their biological function. Several weak intramolecular interactions that stabilize the native conformation exhibit half-lives in the order of the picoseconds to the nanoseconds, implying that those bonds are constantly being formed and broken, giving place to the faster events of protein dynamics^1^. Other common events take place in longer time scales and might have local or global effects over the molecule. Those events, which are a consequence of conformational flexibility, taken together give place to the so-called *protein breathing*.

Such fluctuations are characterized by the temporal time-scale in which they occur (*kinetical* component) and by the magnitude and directionality of the change taking place (*structural* component). It is worth noting that dynamics are closely dependent on the system’s conditions (temperature, pH, ionic strength, buffer components, intermolecular protein−protein interactions, etc.), and that modifying those conditions will result in the alteration of relative populations of states and their interconversion kinetics. The perturbation of global stability, as a consequence of the alteration of the local stability of a region, might result in a change in a molecule’s internal motions. The prediction of the amplitude or the time-scale of the movements that characterize this change is not obvious if the analysis is performed over the 3D structure of the protein alone. To meaningfully address this issue, it is important to acknowledge the differences between global and local events that may govern various phenomena in a protein.

**Figure 1:**
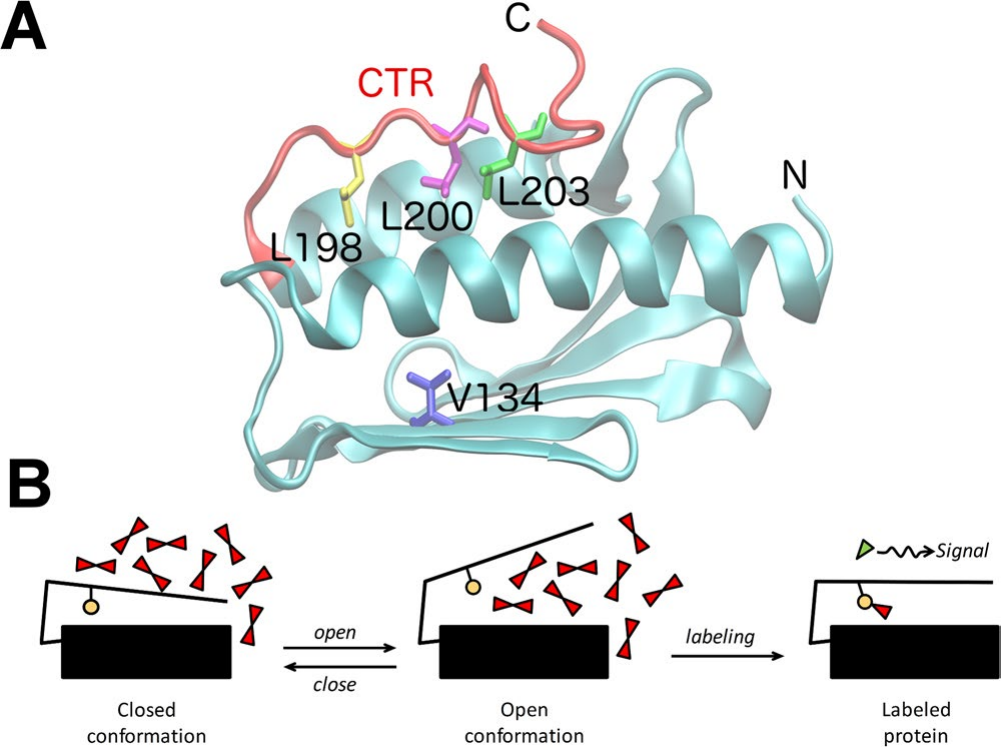
(**A**) Ribbon representation of the structure of human Frataxin (PDB 1EKG). The C-terminal region (CTR) is shown in red and the side-chains of mutated residues are depicted in sticks (L198, L200, L203 and V134 in yellow, magenta, green and blue, respectively). (**B**) Local unfolding of the C-terminal region. Under our working hypothesis, the *open* and *closed* conformations coexist as native substates.

Human Frataxin (FXN) constitutes an excellent model to study the link between protein motions and thermodynamic stability. FXN is a mitochondrial protein, which allosterically activates cysteine desulfurase (NFS1) and promotes the transference of –SH groups from NFS1 to the iron-sulfur cluster assembly enzyme (ISCU). FXN is in the spotlight because its functional deficiency causes a neurodegenerative disease known as Friedreich’s ataxia^2^. Mature human Frataxin (FXN) comprises a single globular domain, composed by a five-stranded antiparallel β-sheet, and two parallel α helices forming an α/β sandwich (Fig. 1A). Residues 196–210 constitute the C-terminal region (CTR), a segment that lacks periodic structure and packs against helices α1 and α2, occluding the apolar side-chains of residues L198, L200, L203 and Y205^3^. While it has non-periodic secondary structure, it is by no means unstructured. This apparently inconsistence between the non-periodic and the structured nature of the CTR, exhibiting high number of interactions with the rest of the protein makes it interesting from a structural dynamic viewpoint. The CTR has been postulated as a key determinant of FXN fold stability^4^. In addition to van der Waals contacts, the CTR establishes a network of electrostatic interactions, including backbone–backbone and side-chain–backbone hydrogen-bonds, with itself and with the rest of the protein. It has been previously shown that the absence of the CTR or a weakened interaction with the compact globular domain affects the dynamics of the protein, leading to destabilized and nonfunctional molecules^5,6^.

Although FXN is markedly resistant to proteolysis, a short peptide involving the last six residues of the CTR is removed by chymotrypsin; residue Y205 is the only proteolysis site found after a 4-hour treatment. This suggests that this part of the stretch is quite mobile; contrastingly, the rest of the domain remains unaltered, revealing its rigidity. Accordingly, and based on a previous report dealing with a truncated variant that lacks the CTR^6^, we hypothesized that local unfolding of this region might allow the modulation of the global structural dynamics and stability by means of an *open–close* mechanism (Fig. 1B), possibly affecting biological functions such as interactions with other proteins or the iron-binding activity.

In the same sense a protein’s *global stability* is determined by the difference in free energy between the folded and unfolded states (${\rm{\Delta }}{G}_{{\rm{N}}\rightleftharpoons {\rm{U}}}^{\circ }$), it is possible to define the *local stability* of a region as the ${\rm{\Delta }}G^\circ$ between the involved states, provided they are in equilibrium. If the difference in free energy between states is small, then it is reasonable to consider that the structural change resulting from *local unfolding* reveals the native dynamics of the protein. A higher difference would account for an intermediate state, while similar values for the global and local ${\rm{\Delta }}G^\circ$ would render their differential analysis unattainable given that local unfolding phenomena would be readily explained by global unfolding.

Thiol-disulfide exchange studies coupled to X → Cys site directed mutagenesis have been applied to map protein topology^7^, to study the native state conformation of the intestinal fatty acid binding protein^8^, and to assess unfolding kinetics, allowing the detection of intermediate states of β-lactamase^9,10^ and tertiary structure disruption of myoglobin^11^. Additionally, thiol-disulfide exchange was applied to evaluate internal motions and local dynamics of myoglobin^12,13^.

In this paper, we address the analysis of the local unfolding phenomena in the CTR of FXN. We present the study of three point-mutants in which a leucine residue, localized in position 198, 200 or 203, is replaced by a cysteine (Fig. 1A). Cysteine residues, which are protected in the *closed* conformation, can be modified by a reactive probe only if exposed to the solvent. In addition, reactivity depends on key-features of the probe as size, hydrophobicity, charge sign and distribution, and nucleophilicity of the thiolate group. The reactivity of a Cys with certain probe depends on the chemical environment and the exposition rate of the thiol to the solvent (probe’s access). This might be a consequence of global or local unfolding events. In the former, the complete molecule is unfolded; in the latter, only a region loses its structure—the CTR in our naive model—while the rest of the molecule retains its native fold. Thiol modification with different reactants enables the study of the mobility (conformational exchange) of diverse positions and the determination of the contribution to stability of the interaction of the CTR with the rest of the molecule. In this way, by applying different stability-modulating conditions, we gained a detailed understanding of the *open*–*close* mechanism, strictly understood as the process by means of which each position fluctuates between an exposed (reactive) and a hidden (non-reactive) conformation.

We found that, despite the lack of periodic structure, the modification rate significantly differs for Cys residues located in different positions along the CTR and the reactivity is significantly lower than the expected for free thiols. Besides, we described the effect over local dynamics of stabilizing or denaturing agents. Based on our results, we propose a reaction mechanism consistent with the existence of at least two native substates.

## Materials and Methods

Please note that while as much methods description as is necessary to follow the logic of the text is detailed below, the bulk of technical description of materials and methods is included in *Supplementary materials*.

### Cysteine modification kinetics

In order to assess the dynamics of specific positions of FXN, we determined proteins’ reactivity towards 5,5′-dithiobis-2-nitrobenzoic acid (DTNB)—also known as Ellman’s reagent—and 4,4′-dithiodipyridine (DTDPy). β-mercaptoethanol (BME) was used to quantify DTNB and DTDPy stock solutions. In order to find the *intrinsic modification* rate coefficient (*k*_*mod*_), we decided to use short peptides corresponding to the stretch comprised between T196 and S206, in which each Leu residue, one at a time, was replaced by a Cys residue. Peptides pL198C: TKCDLSSLAYS, pL200C: TKLDCSSLAYS and pL203C: TKLDLSSCAYS were synthesized (*GenScript*, Piscataway, NJ, USA) and their labeling rates were measured at varying concentrations of DTNB and DTDPy. Proteins or peptides were used at a 30 µM concentration, in a final volume of 500 µl. Solutions were prepared in 20 mM Tris·HCl, 100 mM NaCl, pH 7.0, with varying concentrations of DTNB or DTDPy (0–3.0 mM). Once the probe was added, the solution was gently homogenized and transferred to a cuvette to follow the absorbance change. Time courses were carried out in a Jasco V550 UV/VIS spectrophotometer with a thermostated cell holder connected to a circulating water bath set at 25 °C. For DTNB modification, absorbance was measured at 425 nm, with band-pass of 4 nm, using a 1.0 cm path length cell. For DTDPy modification, absorbance was measured at 324 nm, with band-pass of 4 nm, using a 0.3 cm path length cell. For each kinetic trace, between 1000 and 3000 data points were obtained. When reactions occured in the timescale of seconds, an RX2000 stopped flow instrument (Applied Photophysics Ltd, UK) was used.

### Analysis of local dynamics

In order to describe the local unfolding process, we considered a model that assumes that for a given position, the *open* (reactive) and *closed* (non-reactive) conformations are at equilibrium^14,15^. In such a condition, the concentration of species depends on an *open* microscopic rate coefficient (*k*_*open*_) and a *close* microscopic rate coefficient (*k*_*close*_). In turn, the *open* conformation may react with an appropriate probe (P), with an intrinsic *modification* rate coefficient (*k*_*mod*_) that describes the reaction rate of the thiolate with the reactant. The value of *k*_*mod*_ is the product of the probe’s concentration and the bimolecular rate constant for the reaction of an unprotected free thiol (*k*_*mod*_ = *k* [P]), such as small Cys-containing peptides, BME, cysteine or glutathione. The model can be summarized in the following way:

1 $${Closed} \mbox{-} {\rm{SH}}\,\underset{\mathop{\longleftarrow }\limits_{{k}_{close}}}{\overset{{k}_{open}}{\longrightarrow }}\,Open \mbox{-} {S}^{-}+{\rm{P}}\,\mathop{\longrightarrow }\limits^{\,{k}_{{mod}}\,}\,{Open} \mbox{-} {\rm{S}} \mbox{-} {\rm{label}}+{\rm{R}}$$

The labeling rate depends on the magnitude of the coefficients that make the reaction go forward, toward the *labeled* conformation. If ${k}_{{mod}}\gg {k}_{{close}}$ (EX1 regime), the value of the apparent rate coefficient for the *labeling reaction* (*k*_*label*_) will exclusively depend on *k*_*open*_. On the other hand, if ${k}_{{mod}}\ll {{k}}_{{close}}$ (EX2 regime), *k*_*label*_ will depend on both the equilibrium constant of the *closed*–SH $\rightleftharpoons$ *open*–SH reaction (${K}_{C\rightleftharpoons O}$) and *k*_*mod*_^16^:

2 $$\mathrm{EX1}:\,{{k}}_{{mod}}\gg {k}_{{close}}\,\to {k}_{{label}}={k}_{{open}}$$

3 $$\mathrm{EX2}:\,{{k}}_{{mod}}\ll {{k}}_{{close}}\to {k}_{{label}}={k}_{{mod}}\times {K}_{{\rm{C}}\rightleftharpoons {\rm{O}}}$$

To rule out in which regime the reaction occurs in a certain experimental condition, it suffices to find the dependence of the value of *k*_*label*_ with the concentration of the reactant. If conditions determine an EX1 regime, the value of *k*_*label*_ will be constant given that it only depends on the value of *k*_*open*_, which is independent of the reactant concentration. In the case that the regime is of the EX2 type, the magnitude of *k*_*label*_ will depend on the value of *k*_*mod*_, which varies linearly with the reactant concentration.

Given that in EX2 *k*_*label*_ depends on the values of ${K}_{{\rm{C}}\rightleftharpoons {\rm{O}}}$ and *k*_*mod*_—only dependent on the chemical nature of the reactant and the thiolate—it is possible to determine the local unfolding free energy just by finding the value of the apparent rate coefficient for the labeling reaction. Under steady state conditions *k*_*label*_ may be written in terms of the microscopic rate constants^17^:

4 $${k}_{{label}}=\frac{{{k}}_{{open}}\,{k}_{{mod}}}{{k}_{{open}}+{{k}}_{{close}}+{k}_{{mod}}}$$

Under EX2 conditions (${k}_{{mod}}\ll {{k}}_{{close}}$) *k*_*mod*_ contribution in the denominator may be neglected:

5 $${k}_{label}=\frac{{k}_{open}\,{k}_{mod}}{{k}_{open}+{k}_{close}+{k}_{mod}}\approx \frac{{k}_{open}\,{k}_{mod}}{{k}_{open}+{k}_{close}}$$

On the other hand, a reaction’s equilibrium free energy can be expressed in terms of the constant that defines that equilibrium:

6 $${\rm{\Delta }}{{G}}_{{\rm{C}}\rightleftharpoons {\rm{O}}}^{{\rm{o}}}=-\,{\rm{RT}}\,\mathrm{ln}(\frac{{k}_{{open}}}{{{k}}_{{close}}})$$

The combination of Eqs 5 and 6 gives a relation between the experimentally-measured (apparent) rate coefficient (*k*_*label*_), the *intrinsic microscopic modification* coefficient (*k*_*mod*_) and ${{\rm{\Delta }}G}_{{\rm{C}}\rightleftharpoons {\rm{O}}}^{{\rm{o}}}$

7 $${\rm{\Delta }}{{G}}_{{\rm{C}}\rightleftharpoons {\rm{O}}}^{{\rm{o}}}={\rm{RT}}\,\mathrm{ln}(\frac{{k}_{{mod}}-{k}_{{label}}}{{k}_{{label}}})$$

## Results

### Design of CTR mutants

The alteration of the C-terminal stretch of different FXN homologues has been shown to affect the protein’s global stability^4^. Besides, the effect of point mutations in the CTR and of the complete deletion of this region over the stability, dynamics and folding have been previously studied^5,6,18^. Here we focus on residues L198, L200 and L203, which form a cluster of hydrophobic interactions among themselves and with residues from the core of the protein (Fig. 1A).

Variant FXN90–210, nine residues shorter in its N-terminal region than the mature form FXN81-210 (currently considered the functional form *in vivo*), was the wild-type control sample for conformation and stability, and the base variant over which all mutants were constructed. Note that residues 81–89 have been shown to be disordered^19^. We prepared point-mutants FXN L198C, FXN L200C and FXN L203C, which locate a thiol in different positions of the CTR. Those constructions allowed us to investigate the molecular motions by assessing thiol-exchange reactivity. A fourth mutant, FXN V134C, with its Cys completely protected in the folded ensemble served as a hidden-Cys control.

The four variants are soluble and monomeric, the same as wild-type FXN. Far and near-UV circular dichroism (CD) spectra showed that both the secondary structure content and the tertiary packing are native-like (Fig. S2A,B). Besides, variants’ resistance to proteolysis probed their rigidity (Fig. S3). Moreover, wild-type and Cys point mutants are able to activate desulfurase NFS1 alike, indicating that this function remains intact upon mutation (Fig. S4A). On the other hand, iron binding capability was slightly altered, especially in the case of L200C and in a minor degree in the case of L198C (Fig. S4B).

Solvent accessible surface area and p*K*_a_ predictions performed over computational models based on the X-ray structure of FXN indicated that the mutated residue in each mutant may expose less than 10% of its surface (Fig. S1 and Table S1) and suggested that cysteines would be protonated in a broad range of pH values, particularly in our working conditions. This means that cysteines would mainly behave as apolar residues, capable of establishing most of the hydrophobic interactions that a leucine would establish in the context of the wild-type protein (Fig. S1 and Table S1). However, given the dual polar–apolar nature of cysteine residue, the existence of polar interactions cannot be ruled-out.

### Thermodynamic stability of FXN variants

**Figure 2:**
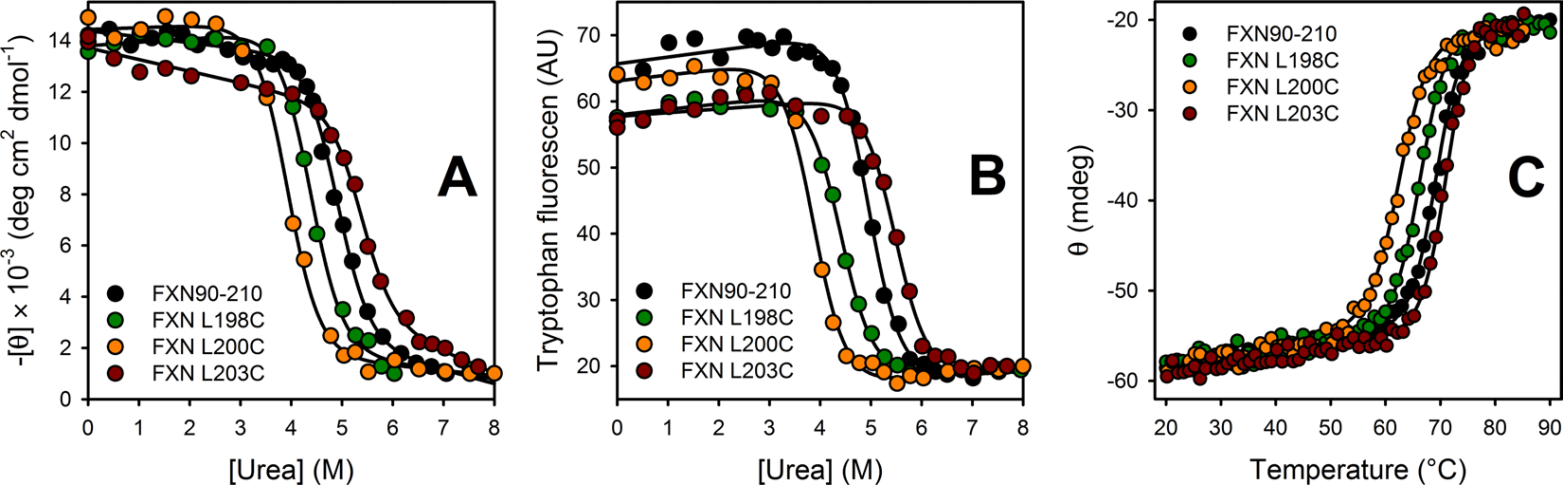
Equilibrium unfolding experiments of FXN Cys-mutants. Urea-induced equilibrium unfolding followed by (**A**) CD at 220 nm and (**B**) tryptophan fluorescence at 335 nm (ex. 295 nm). (**C**) Thermal unfolding followed by CD at 220 nm. Solid lines represent the nonlinear regression fit of the two-state model to the data.

A severe thermodynamic destabilization would preclude us from applying the proposed experimental strategy. For instance, if mutations give rise to very altered equilibrium unfolding constants, a partially folded or unfolded state might be so populated that the labeling reaction could significantly proceed from these states rather that from a substate of N. To investigate whether Cys point mutations affect the native stability of FXN90–210, chemical and temperature equilibrium unfolding experiments were performed (Fig. 2). Although folding kinetic experiments showed that FXN mainly folds via an intermediate state^18^, the shape and overlapping of the unfolding profiles followed by CD and tryptophan fluorescence revealed that all mutants studied in this paper exhibit unfolding curves compatible with a two-state model (${\rm{N}}\rightleftharpoons {\rm{U}}$). This fact was previously described for equilibrium unfolding of several full-length FXN variants^5,6,20^, and it is consequence of the very low population of the I state in equilibrium. Besides, the unfolding reaction is reversible, as judged by signal recovery after temperature-induced unfolding and refolding experiments^18^.

A global fitting of a two-state model, allowing for a unique value for the ${{\rm{m}}}_{{\rm{N}}\rightleftharpoons {\rm{U}}}^{}$ parameter (the dependence of unfolding free energy with denaturant concentration), was performed to experimental data of the three Cys mutants and wild-type FXN (Fig. 2 and Table 1). Our results revealed that Leu → Cys mutations disturb the stability, though not in a great extent. This result is compatible with the fact that the side-chain of cysteine can establish interactions through its −SH group^21,22^. Both *C*_m_ and *T*_m_ decrease in FXN L198C and FXN L200C. On the contrary, a significant increase of *C*_m_ and *T*_m_ is observed for FXN L203C. Mutations L198C and L200C destabilize FXN by ~1 and 2 kcal mol^−1^, respectively, while mutation L203C stabilizes FXN by ~1 kcal mol^−1^.

**Table 1:** Thermodynamic parameters obtained from equilibrium unfolding experiments.

Variant	Urea*						Temperature^†^
	Fluorescence			CD			CD
	${\boldsymbol{\Delta }}{\boldsymbol{\Delta }}{\boldsymbol{G}}{^\circ }_{{\bf{N}}\rightleftharpoons {\bf{U}}}^{{{\bf{H}}}_{{\bf{2}}}{\bf{O}}}$ (kcal mol^−1^)	${\boldsymbol{\Delta }}{\boldsymbol{G}}{^\circ }_{{\bf{N}}\rightleftharpoons {\bf{U}}}^{{{\bf{H}}}_{{\bf{2}}}{\bf{O}}}$ (kcal mol^−1^)	*C*_m_ (M)	${\boldsymbol{\Delta }}{\boldsymbol{\Delta }}{\boldsymbol{G}}{^\circ }_{{\bf{N}}\rightleftharpoons {\bf{U}}}^{{{\bf{H}}}_{{\bf{2}}}{\bf{O}}}$ (kcal mol^−1^)	${\boldsymbol{\Delta }}{\boldsymbol{\Delta }}{\boldsymbol{G}}{^\circ }_{{\bf{N}}\rightleftharpoons {\bf{U}}}^{{{\bf{H}}}_{{\bf{2}}}{\bf{O}}}$ (kcal mol^−1^)	*C*_m_ (M)	*T*_m_ (°C)
FXN90–210	—	09.1 ± 0.5	4.94 ± 0.03	—	9.0 ± 0.5	4.92 ± 0.04	69.4 ± 0.4
FXN L198C	−1.0	08.1 ± 0.5	4.41 ± 0.03	90.9	8.1 ± 0.5	4.42 ± 0.05	66.1 ± 0.3
FXN L200C	−2.0	07.0 ± 0.4	3.85 ± 0.03	−1.8	7.2 ± 0.5	3.95 ± 0.04	62.3 ± 0.3
FXN L203C	−1.0	10.0 ± 0.6	5.47 ± 0.03	−0.8	9.8 ± 0.6	5.35 ± 0.06	71.0 ± 0.4

^*^A two-state model was simultaneously fitted to the data obtained in urea-induced unfolding experiments followed by CD and Trp fluorescence for all variants (Fig. 2A,B). The value of the ${{\rm{m}}}_{{\rm{N}}\rightleftharpoons {\rm{U}}}^{}$ parameter was assumed the same for all variants, and found to be 1.8 ± 0.1 kcal mol^−1^ M^−1^, considerably larger than the value inferred by considering the protein’s length (1.5 kcal mol^−1^ M^−1)^^33^. ${\rm{\Delta }}{\rm{\Delta }}G{^\circ }_{{\rm{N}}\rightleftharpoons {\rm{U}}}^{{{\rm{H}}}_{{\rm{2}}}{\rm{O}}}={\rm{\Delta }}G{^\circ }_{{\rm{N}}\rightleftharpoons {\rm{U}}}^{{{\rm{H}}}_{{\rm{2}}}{\rm{O}},\,\mathrm{wt}}-{\rm{\Delta }}G{^\circ }_{{\rm{N}}\rightleftharpoons {\rm{U}}}^{{{\rm{H}}}_{{\rm{2}}}{\rm{O}},\,\mathrm{mutant}}$. *C*_m_ is the [urea] where 50% of the molecules are unfolded $({\rm{\Delta }}G{{}^{\circ }}_{{\rm{N}}\rightleftharpoons {\rm{U}}}^{{{\rm{C}}}_{{\rm{m}}}^{}}=0)$.

^†^A two-state model was simultaneously fitted to the data obtained in temperature-induced unfolding experiments followed by CD at 220 nm (Fig. 2C). The value of the ${{\rm{\Delta }}C}_{{{\rm{P}}}_{{\rm{N}}\rightleftharpoons {\rm{U}}}}$ parameter—the difference in the heat capacity between the native and unfolded states—was assumed the same for all variants and found to be 1.8 ± 0.3 kcal mol^−1^ K^−1^. *T*_m_ is the temperature where 50% of the molecules are unfolded $({\rm{\Delta }}G{{}^{\circ }}_{{\rm{N}}\rightleftharpoons {\rm{U}}}^{{{\rm{T}}}_{{\rm{m}}}^{}}=0)$.

The thermodynamic stabilization observed for the L203C mutant was unexpected. A possible explanation deduced by visually inspecting the crystallographic structure of FXN is that the introduced cysteine might establish polar interactions with the side chains of S105 and H183, and with atoms from the backbone of L200 (Fig. S5). Besides, the sulfhydryl group may also be involved in stabilizing electrostatic interactions such as hydrogen bonds, possibly leading to the alteration of the p*K*_a_ of that group^21^, in agreement with molecular dynamic simulation results (data not shown).

The dependence of stability on pH was also analyzed. No significant effect of the pH in the range 6.0–8.0 was detected, suggesting that the population of molecules with their cysteine’s thiol in the thiolate form is negligible, even at pH 8.0 (Fig. S6 and Table S2). This result is consistent with *in silico* p*K*_a_ predictions (Table S1), and allows us infer that in our regular working conditions the cysteine of the three variants is protonated and would consequently behave as a hydrophobic residue. This supports the idea that cysteine residues have the capability of satisfying to a good extent the original interactions established by leucine in wild-type FXN.

### Cysteine residue modification

**Figure 3:**
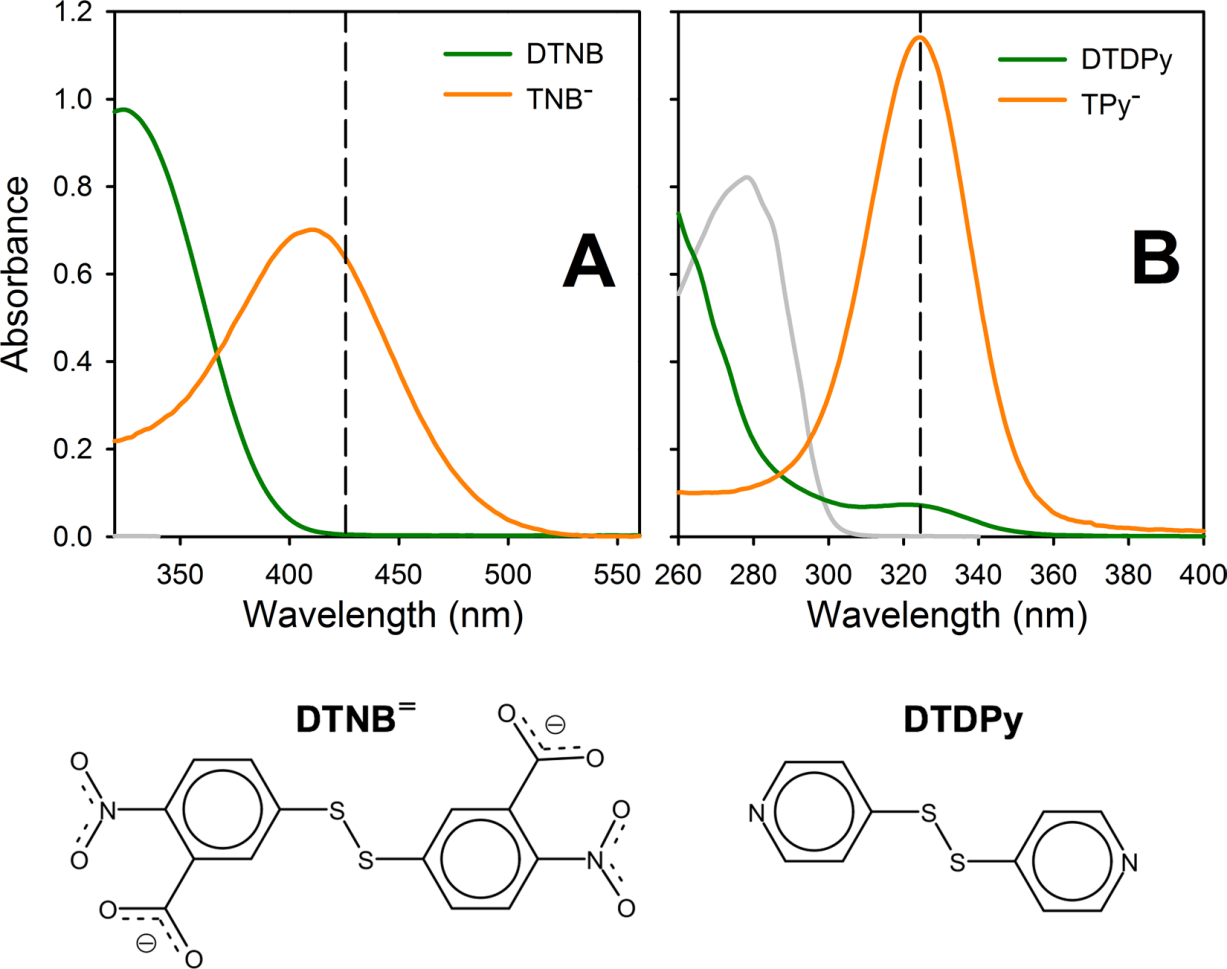
Probes used to evaluate cysteine accessibility. Absorption spectrum and chemical structure of (**A**) DTNB and ${{\rm{TNB}}}^{=}$ (50 µM), and (**B**) DTDPy and TPy^−^ (50 µM). TNB^=^ and ${{\rm{TPy}}}^{-}$ were generated by the addition of excess BME to 25 µM DTNB and DTDPy, respectively. FXN (30 μM) absorption spectrum (gray) is included to show protein absorption contribution to the measured signal. Vertical lines mark the wavelengths used to follow chemical modification reactions: 425 nm for DTNB and 324 nm for DTDPy. Ellman’s reagent or DTNB: 5,5′-dithiobis-2-nitrobenzoic acid and DTDPy: 4,4′-dithiodipyridine.

In order to estimate the dynamics of the C-terminal region, we used two reactive probes with different size and charge: 5,5′-dithiobis-2-nitrobenzoic acid (DTNB) and 4,4′-dithiodipyridine (DTDPy). Each probe is composed by two identical aromatic rings, linked by a disulfide (Fig. 3). DTNB consists of two molecules of 2-nitro-5-thiobenzoic acid, linked through position 5. DTDPy consists of two pyridines linked through position 4. A free sulfhydryl group, in its thiolate form can react with a molecule of DTNB or DTDPy to produce a mixed disulfide (labeled protein) and a molecule of 5-thio-2-nitrobenzoic acid (${{\rm{TNB}}}^{=}$) or 4-thiopyridine (${{\rm{TPy}}}^{-}$), respectively:

8 $${{\rm{R}}-{\rm{S}}}^{-}+{{\rm{DTNB}}}^{=}\to {{\rm{R}} \mbox{-} {\rm{S}} \mbox{-} {\rm{S}} \mbox{-} {\rm{TNB}}}^{-}+{{\rm{TNB}}}^{=}$$

9 $${{\rm{R}}-{\rm{S}}}^{-}+{\rm{DTDPy}}\to {\rm{R}} \mbox{-} {\rm{S}} \mbox{-} {\rm{S}} \mbox{-} {\rm{TPy}}+{{\rm{TPy}}}^{-}$$

Note that in the case of FXN, the formation of dimers of the form FXN-S-S-FXN seems rather improbable, given we have never observed dimers despite not having treated the protein with any reducing agent. Besides, the effective concentration of free thiols that should be consider in order to establish the magnitude of probe’s excess is that of the protein in which the Cys residue is in the *open* (solvent accessible) conformation, which is small in all our variants, as discussed later.

The reaction progress is readily followed given that the hemimolecules of both probes possess different absorption spectra than the original molecule (Fig. 3). Besides, at the specific wavelengths that we used to follow the reactions (425 nm for DTNB and 324 nm for DTDPy), there is no significant protein absorption.

### Kinetics of cysteine modification

**Figure 4:**
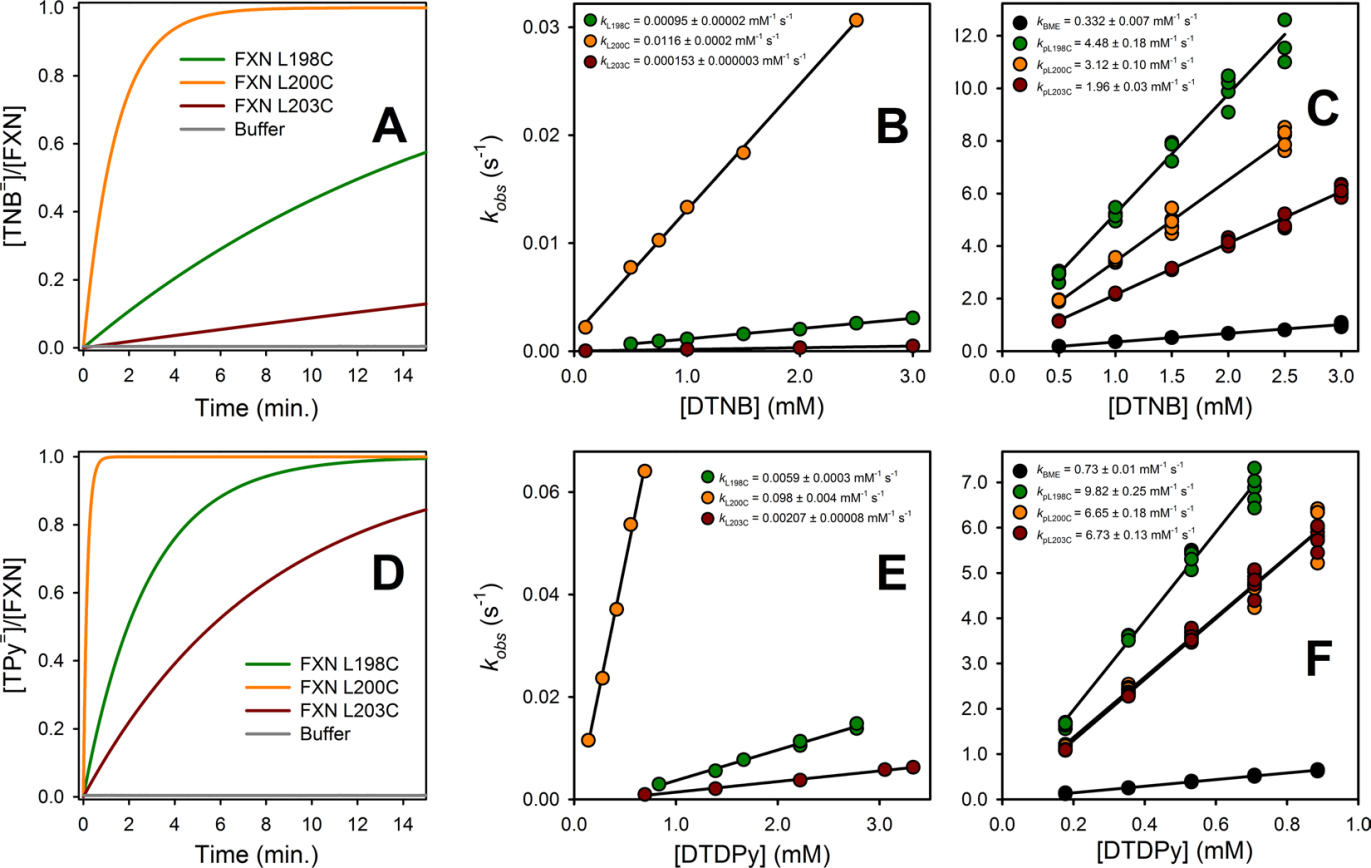
Cysteine modification experiments. The reactivity of FXN Cys-mutants and Cys-peptides was determined for DTNB (**A–C**) and DTDPy (**D**–**F**). (**A**,**D**) show the time traces of the reactions with 1 mM DTNB (**A**) or DTDPy (**D**). Similar curves were obtained at different probe concentrations. A monoexponential function of time was fitted to the data in order to find *k*_*label*_ for each mutant (**B** and **E**). The reaction intrinsic constant, *k*_*mod*_, was obtained from a similar fitting to the Cys-peptides’ curves (C and F). We obtained the concentration-independent second-order kinetic rate constants from the linear regression of pseudo-first order constants found each probe concentration (B, C, E and F).

Reactivity toward DTNB and DTDPy was analyzed. Cys mutants show significant differences in the observed rate coefficients values (*k*_*label*_), being FXN L200C the most reactive and FXN L203C the one with the lowest modification rate (Fig. 4). The three mutants are much less reactive than their respective peptides (pL198C, pL200C or pL203C) or BME, indicating that there exists some degree of protection of the thiol toward chemical modification, irrespectively of the position of the Cys residue. Furthermore, given that the modification reaction effectively occurs, there must exist some dynamic process that allows the sulfhydryl group to be exposed only during a fraction of the time. In addition, given the molecular size of DTNB and DTDPy (van der Waals surface areas 434 and 277 Å^2^, respectively), a significant extent of Cys exposition would be necessary for the probe to react.

Interestingly, the three peptides are significantly more reactive than BME and significant differences in reactivity are observed among them. Given that the peptides are only 11-residue long, this result suggests that the delicate modulation of the thiol reactivity depends on the very local molecular environment of the Cys residue. In particular, Lys2 gives the peptide a net positive charge in the N-terminal region; considering the proximity between the Lys and the Cys residue, it may be expected that reactivity of pL198C > pL200C > pL203C^22^, coincidently with our experimental observations. Further experiments will be carried out in order to establish the differences in p*K*a and/or nucleophilicity of the thiol for each peptide. It is worthy to mention that the use of small molecules like BME, glutathione or even cysteine should be used with caution for this kind of studies.

**Figure 5:**
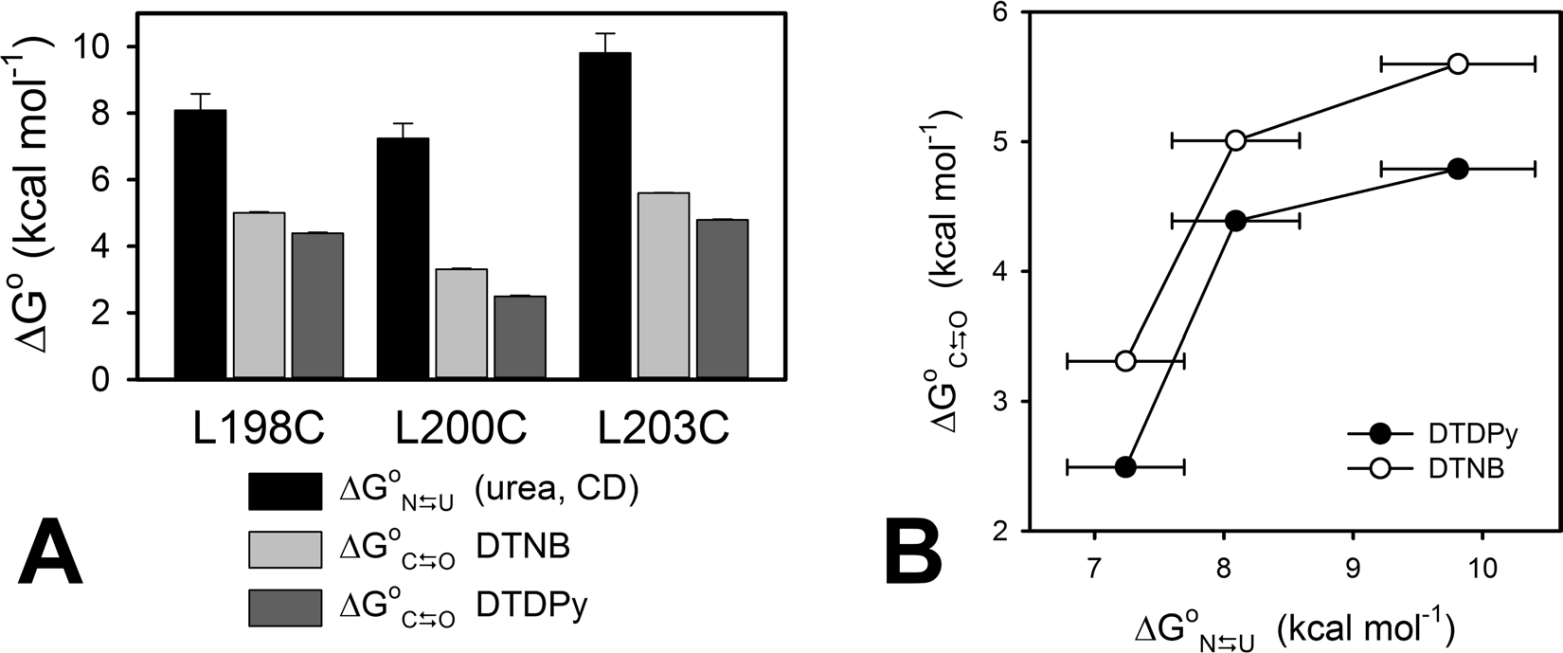
Global and local stability of FXN Cys-mutants. (**A**) ${\rm{\Delta }}{G}_{{\rm{N}}\rightleftharpoons {\rm{U}}}^{{\rm{o}}}$ values obtained from urea-induced equilibrium unfolding experiments followed by CD at 220 nm (Table 1), and ${\rm{\Delta }}{G}_{{\rm{C}}\rightleftharpoons {\rm{O}}}^{{\rm{o}}}$ values obtained with DTNB and DTDPy. Error bars show ± 1 S.E of the fitting to the two-state model (global unfolding, ${\rm{\Delta }}{G}_{{\rm{N}}\rightleftharpoons {\rm{U}}}^{{\rm{o}}}$), or ± 1 S.E of the linear regression of ${k}_{{mod}}$ and ${k}_{{label}}$ as a function of the probe’s concentration (Fig. 4 B, C, E and F), propagated in the free energy calculation (local unfolding, ${\rm{\Delta }}{G}_{{\rm{C}}\rightleftharpoons {\rm{O}}}^{{\rm{o}}}$ DTNB and ${\rm{\Delta }}{G}_{{\rm{C}}\rightleftharpoons {\rm{O}}}^{{\rm{o}}}$ DTDPy). (**B**) Local stability dependence on global stability (${\rm{\Delta }}{G}_{{\rm{C}}\rightleftharpoons {\rm{O}}}^{{\rm{o}}}$ vs. ${\rm{\Delta }}{G}_{{\rm{N}}\rightleftharpoons {\rm{U}}}^{{\rm{o}}}$).

Values corresponding to the observed labeling coefficients, *k*_*label*_, were obtained for each Cys mutant at varying probe concentration. Although knowing *k*_*mod*_ and *k*_*label*_ at an identical reagent concentration would suffice to calculate the free energy associated to the CTR’s *open-close* process (Equation 7), *k*_*label*_ dependence on the reagent’s concentration is necessary to establish the regime under which the reaction occurs. Moreover, given this is a bimolecular reaction, the rate of change of *k*_*label*_ with the change in the concentration of the probe provides its concentration-independent value, which is a more accurate value as it is the result of the regression to multiple experimental data. The same criteria apply for the determination of the intrinsic modification coefficient, *k*_*mod*_ for BME and the three model peptides pL198C, pL200C and pL203C (Fig. 4C,F). Obtained concentration-independent values of *k*_*label*_ and *k*_*mod*_ were used to calculate the value of Δ*G*° for the *open–close* reaction (Fig. 5 and Table 2). Additionally, the comparison between Δ*G*° values for the *open–close* process obtained using the *k*_mod_ values for BME or peptide models is shown Fig. S7 (Supplementary Material).

**Table 2:** Local (${\rm{C}}\rightleftharpoons {\rm{O}}$) and global (${\rm{N}}\rightleftharpoons {\rm{U}}$) unfolding equilibrium constants.

		FXN L198C	FXN L200C	FXN L203C
${\rm{C}}{\rm{\rightleftharpoons }}{\rm{O}}$ equilibrium DTNB	${\rm{\Delta }}{{G}}_{{\rm{C}}\rightleftharpoons {\rm{O}}}^{{\rm{o}}}$	5.01 ± 0.03	3.31 ± 0.02	5.60 ± 0.01
	${{K}}_{{\rm{C}}\rightleftharpoons {\rm{O}}}$	0.00021 ± 0.00001	0.0037 ± 0.0001	0.000078 ± 0.000002
	f_C_/f_O_^†^	4 704 ± 207	267 ± 10	12 760 ± 303
${\rm{C}}{\rm{\rightleftharpoons }}{\rm{O}}$ equilibrium DTDPy	${\rm{\Delta }}{{G}}_{{\rm{C}}\rightleftharpoons {\rm{O}}}^{{\rm{o}}}$	4.39 ± 0.03	2.49 ± 0.03	4.79 ± 0.03
	${{K}}_{{\rm{C}}\rightleftharpoons {\rm{O}}}$	0.00061 ± 0.00003	0.0149 ± 0.0007	0.000308 ± 0.000014
	f_C_/f_O_^†^	1652 ± 84	67 ± 3	3252 ± 144
${\rm{N}}{\rm{\rightleftharpoons }}{\rm{U}}$ equilibrium Urea, CD	${\rm{\Delta }}{{G}}_{{\rm{N}}\rightleftharpoons {\rm{U}}}^{{\rm{o}}}$	8.1 ± 0.5	7.2 ± 0.5	9.8 ± 0.6
	${{K}}_{{\rm{N}}\rightleftharpoons {\rm{U}}}$^*^	0.0000012 ± 0.0000004	0.000005 ± 0.000001	0.00000006 ± 0.00000003
	f_N_/f_U_^†^	859 672	204 283	15 677 417

^†^The *closed* (C) to *open* (O) ratio and the native (N) to unfolded (U) ratio are the inverse value of the corresponding equilibrium constant.

^*^Equilibrium constants were obtained by fitting a two-state model to the urea-induced unfolding experiments followed by CD (Fig. 2A and Table 1).

Δ*G*° values for the *open–close* reaction indicate that local conformational changes are occurring, given that the difference in free energy associated to the global unfolding process (${\rm{N}}\rightleftharpoons {\rm{U}}$) is considerably larger (Fig. 5). In the same vein, FXN L134C shows a lower reactivity than any of the CTR mutants, despite being even less stable than FXN L200C. In fact, the difference in Δ*G*° for the *open*–*close* reaction is 5.3 ± 0.2 kcal mol^−1^ in this case (calculated by considering the *k*_mod_ corresponding to the BME thiol), a value that is compatible with the complete unfolding of the protein.

## Discussion

The complete understanding of protein function cannot be obtained from the mere analyses of the static three-dimensional structure. Instead, it requires a deep knowledge of the native state structural dynamics, given that function tightly depends on the molecule’s internal mobility. Although the analyses based on X-ray diffraction or NMR solution structures may provide good information—particularly about spatial localization of reactive residues, interaction regions y ligand-binding sites—the study of the temporal courses, frequencies and amplitudes of intramolecular movements and fluctuations is essential in order to understand biological processes and minutely characterize function-related native substates. Experimental access to the temporal dimension and high-resolution study of substates at the secondary structure and amino acid residue level is a challenging task. One of the most popular tools used to investigate internal mobility and activated states is NMR relaxation dispersion. Besides, hydrogen–deuterium exchange allows the study of slow processes (occurring in the milliseconds and seconds time scales) and the determination of equilibrium constants of local events, but relies on auxiliary techniques such as NMR or mass spectroscopy for the identification of the residues involved in exchange processes^1^.

The fact that certain regions may experience local unfolding events that do not imply the global unfolding of the protein, suggests the existence of several minimal-energy substates: the native state ensemble. That kind of events have been described in several proteins that depend on local unfolding for a ligand to enter a cavity, such as retinol-binding protein^23^, the interaction with the active site, like in transglutaminase^24^, enzymatic catalysis itself, such as in peroxirredoxines^25^, and even as a determinant of the degradation rate^26^ or of the capability to aggregate as amyloid fibrils^27^. It was also suggested that defencins—a group of short, cationic, cysteine-rich, amphiphilic peptides, capable of neutralizing several different exotoxins—may exert their action mechanism by inducing local unfolding of certain regions of several microbial and viral proteins^28^.

In this work, we studied the reactivity of sulfhydryl groups introduced in positions 198, 200 and 203 of FXN to explore the effect of the alteration of the interaction network established by the CTR of the molecule. By means of the analysis of the differential reactivity we characterized the dynamics of each position. Besides, we could calculate the change in free energy associated with the *closed* $\rightleftharpoons$ *open* reaction in order to determine the energetics of the process involving those fluctuations.

A cysteine’s reactivity depends on its thiol group nucleophilicity, its accessibility to the solvent (exposition rate) and its ionization state, which is modulated by the chemical environment (e.g. ionic interactions with neighbor residues), being the thiolate state (R − S^−^) the one that may react. Naturally, a hidden, completely buried cysteine will have no access to the probe dampening the reactivity of the *closed* conformation (Fig. 1B). The apolar environment provided by the hydrophobic core favors the less reactive thiol state (R − SH). Instead, when the sulfhydryl group is exposed to the solvent (*open* conformation) its deprotonation will be a more probable event.

In wild-type FXN, L198, L200 and L203 contribute to the stability of the interaction between the core and the CTR^3,4^. The substitution of leucine for cysteine produces the shortening of the side-chain by two methyl groups and replaces the Cγ for an S atom. Such a modification necessarily implies the loss of interactions, what may destabilize the molecule^6^. However, given the strong apolar character of a protonated thiol^29^, it is possible for the original interactions established by leucine to partially prevail. In fact, p*K*_a_ values are predicted higher than expected for a free solvated thiol (Table S1), and in agreement with this prediction, the conformational stability of none the Cys mutants changed significantly in response to a change in pH (Fig. S6).

The intrinsic microscopic modification coefficient (*k*_mod_) for each mutant was evaluated using the synthetic peptides pL198C, pL200C and pL203C, which mimic the chemical environment of the corresponding Cys residue when the C-terminal region is unfolded (*open* state). Our results showed that BME—a completely-exposed thiol of low molecular weight—reacts significantly slower than the peptides, suggesting that reactivity is modulated by the peptidic environment under our experimental conditions. For model peptides, significant shifts of thiol p*K*a values may be explained by the presence of positively charged residues near the Cys side chain^22^. On the other hand, we cannot rule out a potential overestimation of the reactivity of these peptides as the result of the proximity of the N-terminus amine group. In this regard, the N-acetylation of the peptides might contribute to solve this issue.

DTNB exhibited significantly lower modification rates than DTDPy. Consequently, ${\rm{\Delta }}{G}_{{\rm{C}}\rightleftharpoons {\rm{O}}}^{{\rm{o}}}$ values obtained for DTDPy were lower than for DTNB. However, the modification rates of the three Cys mutants followed the same pattern for both reagents. DTNB is charged and much voluminous a molecule than DTDPy, which consists in just two pyridine rings linked by a disulfide bond (Fig. 3). Therefore, DTDPy may have an easier access to partially exposed thiols, being able to detect fluctuations that imply a smaller structural change. In the same fashion, Stratton, *et al*.^13^ showed that the kinetic of thiol-disulfide exchange involving Cys residues located at specific positions in myoglobin was highly dependent on the size of the thiosulfonate-probe reagent.

### Cys exposition rate is only explainable by local fluctuations

The accessibility of probes to the thiolate might well be explained by global unfolding events. In the case of a protein that unfolds via a two-state mechanism, those events occur according to the equilibrium constant of the ${\rm{N}}\rightleftharpoons {\rm{U}}$ reaction, ${K}_{{\rm{N}}\rightleftharpoons {\rm{U}}}$. Such is the case of variant FXN V134C, which has a Cys residue located deep in the core of the protein, only accessible when the molecule unfolds. In native conditions, the fraction of molecules in the U state is several orders of magnitude lower than in the N state. Given that its reactivity relies on global unfolding, and is thus related to the global conformational stability, FXN V134C reacted extremely slowly.

The case of CTR Cys-mutants is contrasting. As derived from the calculated equilibrium constants (Table 2), it is not possible to explain cysteine modification just by global unfolding events, given that ${K}_{{\rm{N}}\rightleftharpoons {\rm{U}}}$ is two, three and three orders of magnitude lower than the equilibrium constant of the ${\rm{C}}\rightleftharpoons {\rm{O}}$ reaction, ${K}_{{\rm{C}}\rightleftharpoons {\rm{O}}}$, for the L198C, L200C and L203C, respectively.

For a ballpark analysis, let us consider that the dynamics of the CTR in wild-type FXN is somewhere in between the behavior of mutants FXN L198C and FXN L203C. The difference in free energy between the *open* and *closed* forms dictates that every 10^4^ molecules, less than 2 exist in the *open* state (when the mechanism is studied by means of DTNB reactivity). This means that just a fraction of native molecules would exhibit the *open* conformation, while a much higher number of molecules would have the CTR packed against the rest of the protein (Table 2).

### A comprehensive model for cysteine exposition

**Figure 6:**
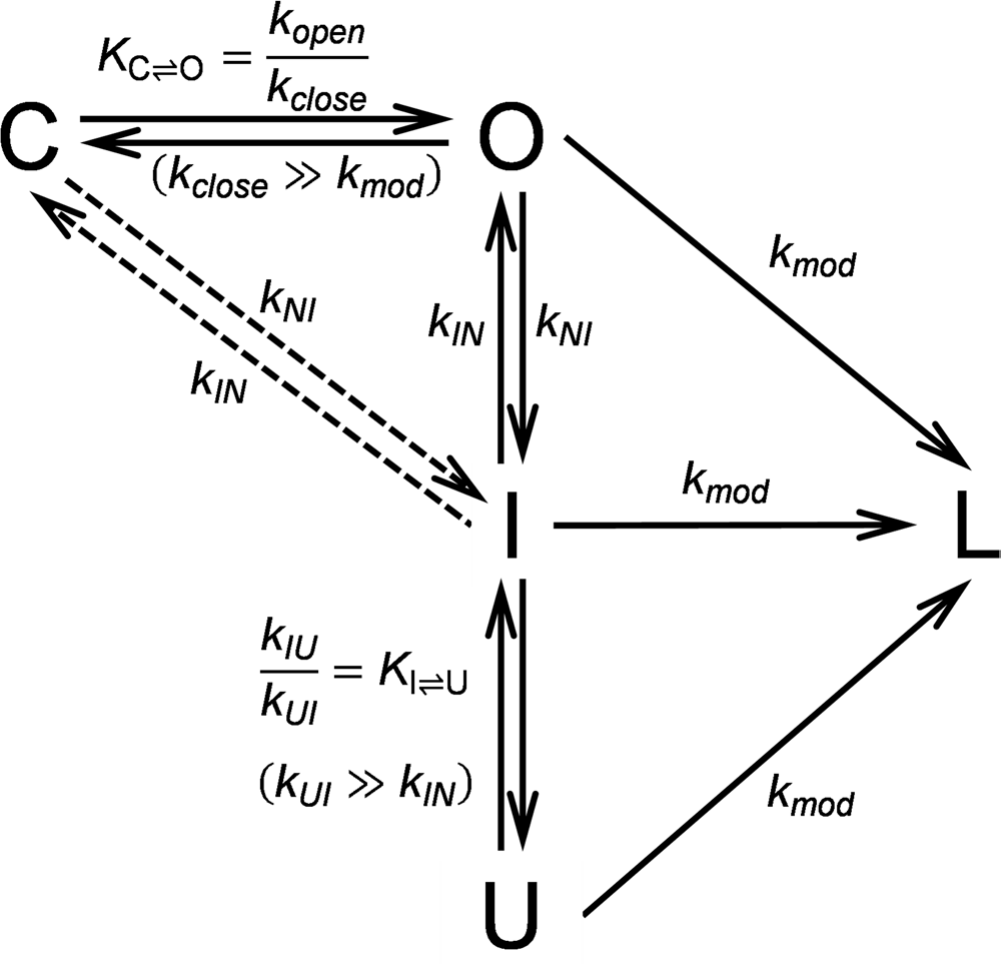
Scheme of conformational fluctuations of human Frataxin. FXN folds through an intermediate state (I) with low compactness and ~60% of native secondary structure. Our working hypothesis states that there exist at least two native substates: a *closed* conformation (C), in which the CTR residues are hidden, and an *open* conformation (O), in which the CTR possesses a higher solvent accessible surface area and may react to produce the labeled protein (L). Both the *closed* and the *open* conformations are native forms of the protein, different from the intermediate and unfolded (U) state. However, the same as the *open* conformation, the intermediate state—which has an unstructured C terminal^18^—and the unfolded state may react with the probes. The *open* native state may turn to the intermediate and unfolded states. Besides, the *closed* native state may directly turn to the intermediate (dashed arrow).

In order to challenge the robustness of our simplified analytical approach, and to account for contributions of global unfolding processes to the apparent modification rate, we proposed a more complex model which includes the intermediate state (Fig. 6), only detectable by means of rapid-mixing kinetic studies^18^. This consideration is particularly relevant, given that the CTR is not structured in that state^18^. Therefore, a cysteine located in that region would be just as reactive in the intermediate (I) state as it would be in the unfolded (U) state. Consequently, the observed modification rate may well be explained by the ${\rm{N}}\rightleftharpoons {\rm{I}}$ interconversion, and there would be no need of invoking local unfolding phenomena of molecules in the N state (${\rm{C}}\rightleftharpoons {\rm{O}}$) in order to rationalize our results. Furthermore, we wondered if fitting the complete model to the data would result in better estimation of equilibrium constants than what is obtained by applying the simplified treatment of data resulting in Eq. 7. The proposed model (Fig. 6) was simultaneously fitted to chemical modification time courses performed at varying probe concentration. The model adequately describes the results (Fig. S9) for the best fitting values of the parameters listed in Table 3. The equilibrium constant was systematically reproduced in several fittings with varying starting values of the parameters. The values obtained for ${K}_{{\rm{C}}\rightleftharpoons {\rm{O}}}$ coincided with those calculated using our simplified procedure, reinforcing its validity.

**Table 3:** Best-fitting values of the parameters of the model.

Parameter	FXN L198C	FXN L200C	FXN L203C
*k*_*open*_ (s^−1^)	0.0278 ± 0.003	0.0372 ± 0.0002	0.04 ± 0.01^*^
*k*_*close*_ (s^−1^)	137 ± 2x	6.89 ± 0.07	608 ± 167^*^
${{K}}_{{\bf{C}}\rightleftharpoons {\bf{O}}}$	2.0 × 10^−4^	5.4 × 10^−3^	6.6 × 10^−5^
*k*_*mod*_ (mM^−1^ s^−1^)	5.38 ± 0.02	3.55 ± 0.03	2.182 ± 0.008

The model presented in Fig. 6 was fitted simultaneously to DTNB modification time courses of each Cys-mutant and their corresponding Cys-peptide using COPASI^34^. Initial values were those corresponding to the equilibrium distribution of species in the absence of probe. Parameters are presented as value ± 1 S.E.

Fixed parameter values were the following^18^: *k*_*IN*_: 24.9 s^−1^, *k*_*UI*_: 249 s^−1^ (${k}_{{UI}}={\rm{10}}\times {k}_{{IN}}$) and *k*_*IU*_: 8 s^−1^ (${K}_{{\rm{I}}\rightleftharpoons {\rm{U}}}$: 0.03); for FXN L198C, *k*_*NI*_: 9 × 10^−4^ s^−1^ (${K}_{{\rm{N}}\rightleftharpoons {\rm{I}}}$: 4 × 10^−5^); for FXN L200C, *k*_*NI*_: 0.004 s^−1^ (${K}_{{\rm{N}}\rightleftharpoons {\rm{I}}}$: 2 × 10^−4^); for FXN L203C, *k*_*NI*_: 5 × 10^−5^ s^−1^ (${K}_{{\rm{N}}\rightleftharpoons {\rm{I}}}$: 2 × 10^−6^).

^*^The fitting of FXN L203C data was undetermined: other values for *k*_*open*_ and *k*_*close*_ are equally probable, provided the quotient between those parameters (${K}_{{\rm{C}}\rightleftharpoons {\rm{O}}}$) does not vary.

The numerical fitting of the model allows us to analyze the contribution of the rate coefficients to the ${K}_{{\rm{C}}\rightleftharpoons {\rm{O}}}$. It is interesting that the most notorious variation is observed for the values of *k*_*close*_. Not only are *k*_*close*_ values more distinct between different mutants, but also, they are between one and four orders of magnitude higher than *k*_*open*_. This suggests that the local fluctuation and the correlated global stability are heavily dependent on the *local refolding* process, adding a complementary explanation to the *conformational lock* hypothesis^6^.

Simulations were useful to determine if it is correct to assume a monoexponential behavior (governed by a unique observed rate coefficient) of the labeling process (Fig. S8). We have described that if experimental conditions are those that guarantee the reaction to occur under an EX2 regime, it is possible to simplify equations enough so that the global reaction might be consider a first-order one. Given that a monoexponential function of time can be correctly fitted to the result of the simulations of the labeling reaction (still obeying the model shown in Fig. 6), it is evident that such an equation accurately explains the data (Fig. S8B).

It is noteworthy that our analysis shed light on the timescale in which the conformational changes of the studied process occurs. The inferred values for the microscopic coefficients *k*_*open*_ indicate that these conformational changes are seemingly infrequent. Assuming a good correlation between global free energy of unfolding and *k*_close_, one may infer the corresponding *k*_*close*_ value for wild-type FXN to be ∼47 s^−1^. Cys-modification analysis allowed us to detect motions that were not previously detected by NMR. The *T*_1_*/T*_2_ ratio values suggest no enhanced relaxation in the CTR under native conditions and CPMG relaxation dispersion experiments show that this stretch of wild-type FXN does not significantly contribute to the protein chemical/conformational exchange^5,30^. Although such observations are apparently in conflict with Cys-modification analysis and with results that suggest that Y205 (located in the CTR) is the first proteolytic site for wild-type FXN after a long incubation with chymotrypsin^6^, they are not unexpected given that those NMR experiments explores conformational dynamics in the pico to milliseconds time scales. Remarkably, the fluctuations involving the CTR in our working model are consistent with processes occurring in the order of seconds to minutes (Table 3).

One may consider that the differences in reactivity found for our mutants might be the result of very large perturbations of the hydrophobic interactions occurring between the CTR and the *core* of FXN; consequently, the behavior observed for this set of mutants might not be illustrative of the motions and local unfolding events that exist in wild-type FXN. However, these mutants are quite stable from the thermodynamic viewpoint, revealing that the interactions that CTR establishes are substantially preserved. This fact, added to the finding of stabilizing (L203C) and destabilizing mutations (L198C and L200C), and to the existence of a trend between global and local stabilities, might allow us to estimate local unfolding and CTR dynamics of wild-type FXN, therefore gaining significant information about its native motions.

Finally, our results indicate that there is a relationship between local stability of the CTR and global stability of FXN and this is consistent with the strong loss of stability and increase of mobility of the CTR seen in the pathogenic variant FXN L198R^5^. It has been shown that a number of pathogenic mutations that produce Friedreich’s ataxia involves protein variants that are highly destabilized showing significantly lower melting temperatures and lower free energy of unfolding by comparison to the wild-type FXN, among them: G130V (Δ*T*_m_ = 23 °C and ΔΔ*G*°_NU_ ∼2.9 kcal mol^−1^)^31^, G137V (Δ*T*_m_ ~20 °C)^32^ and L198R (Δ*T*_m_ = 15.3 °C and ΔΔ*G*°_NU_ ∼4.2 kcal mol^−1^)^5^. Local stabilization of the CTR in these cases may produce an increase in global stability of the mutant proteins. This means that the CTR may be a promising target for drug design and therapeutic strategies.

### Concluding remarks

In this paper, we addressed the study of local fluctuations, derived from native state dynamics, by means of the analysis of the reactivity of cysteine in a set of rationally-designed point mutants. We proved the existence of local conformational changes, which imply the exposition of the side-chains of residues that are relevant for global stability and are hidden from the solvent in the most populated native substate. Furthermore, we could get some insight into the energetics of that local process and quantified the local stability of FXN’s CTR for each of the mutants. Our results show that chemical modification can be efficiently used to assess the existence of native substates that characterize the dynamics and affect stability of protein molecules.

## Supplementary information

Supplementary Information
