# Author Correction: Global Implications of Local Unfolding Phenomena, Probed by Cysteine Reactivity in Human Frataxin

**DOI:** 10.1038/s41598-020-57572-z

**Published:** 2020-01-14

**Authors:** Santiago E. Faraj, Martín E. Noguera, José María Delfino, Javier Santos

**Affiliations:** 1 Alejandro Paladini Institute of Biological Chemistry and Chemical Physics (UBA-CONICET), Faculty of Pharmacy and Biochemistry, University of Buenos Aires, Junín 956, (C1113AAD), Buenos Aires, Argentina; 2 Departamento de Fisiología y Biología Molecular y Celular, Facultad de Ciencia Exactas y Naturales, Universidad de Buenos Aires. Instituto de Biociencias, Biotecnología y Biomedicina (iB3). Intendente Güiraldes 2160 - Ciudad Universitaria, 1428EGA C.A.B.A., Argentina

Correction to: *Scientific Reports* 10.1038/s41598-019-39429-2, published online 11 February 2019

The Article contains errors in Table 1, where the reported second column heading under Urea CD was incorrectly titled. Additionally, there are errors in four of the values listed under ‘Urea Fluorescence’ and ‘Urea CD’.

The correct Table 1 is shown below.

**Table 1:** Thermodynamic parameters obtained from equilibrium unfolding experiments.

Variant	Urea*						Temperature^†^
	Fluorescence			CD			CD
	${\boldsymbol{\Delta }}{\boldsymbol{\Delta }}{\boldsymbol{G}}{{\boldsymbol{^\circ }}}_{{\bf{N}}\rightleftharpoons {\bf{U}}}^{{{\bf{H}}}_{{\bf{2}}}{\bf{O}}}$ (kcal mol^−1^)	${\boldsymbol{\Delta }}{\boldsymbol{G}}{{\boldsymbol{^\circ }}}_{{\bf{N}}\rightleftharpoons {\bf{U}}}^{{{\bf{H}}}_{{\bf{2}}}{\bf{O}}}$ (kcal mol^−1^)	C_m_ (M)	${\boldsymbol{\Delta }}{\boldsymbol{\Delta }}{\boldsymbol{G}}{{\boldsymbol{^\circ }}}_{{\bf{N}}\rightleftharpoons {\bf{U}}}^{{{\bf{H}}}_{{\bf{2}}}{\bf{O}}}$ (kcal mol^−1^)	${\boldsymbol{\Delta }}{\boldsymbol{G}}{{\boldsymbol{^\circ }}}_{{\bf{N}}\rightleftharpoons {\bf{U}}}^{{{\bf{H}}}_{{\bf{2}}}{\bf{O}}}$ (kcal mol^−1^)	C_m_ (M)	T_m_ (°C)
FXN90-210	—	9.1 ± 0.5	4.94 ± 0.03	—	9.0 ± 0.5	4.92 ± 0.04	69.4 ± 0.4
FXN L198C	1.0	8.1 ± 0.5	4.41 ± 0.03	0.9	8.1 ± 0.5	4.42 ± 0.05	66.1 ± 0.3
FXN L200C	2.0	7.0 ± 0.4	3.85 ± 0.03	1.8	7.2 ± 0.5	3.95 ± 0.04	62.3 ± 0.3
FXN L203C	−1.0	10.0 ± 0.6	5.47 ± 0.03	−0.8	9.8 ± 0.6	5.35 ± 0.06	71.0 ± 0.4

*A two-state model was simultaneously fitted to the data obtained in urea-induced unfolding experiments followed by CD and Trp fluorescence for all variants (Figure 2A and B). The value of the ${{\rm{m}}}_{{\rm{N}}\rightleftharpoons {\rm{U}}}^{.}$ parameter was assumed the same for all variants, and found to be 1.8 ± 0.1 kcal mol^−1^ M^−1^, considerably larger than the value inferred by considering the protein length (1.5 kcal mol^−1^ M^−1^)^1^. $\Delta \Delta G{^\circ }_{{\rm{N}}\rightleftharpoons {\rm{U}}}^{{{\rm{H}}}_{2}{\rm{O}}}=\Delta G{^\circ }_{{\rm{N}}\rightleftharpoons {\rm{U}}}^{{{\rm{H}}}_{2}{\rm{O}},{\rm{wt}}}-\Delta G{^\circ }_{{\rm{N}}\rightleftharpoons {\rm{U}}}^{{{\rm{H}}}_{2}{\rm{O}},{\rm{mutant}}}$. C_m_ is the [urea] at which 50 % of the molecules are unfolded $(\Delta G{^\circ }_{{\rm{N}}\rightleftharpoons {\rm{U}}}^{{{\rm{C}}}_{m}}=0)$.

^†^A two-state model was simultaneously fitted to the data obtained in temperature-induced unfolding experiments followed by CD at 220 nm (Figure 2C). The value of the ${\Delta C}_{{{\rm{P}}}_{{\rm{N}}\rightleftharpoons {\rm{U}}}}$ parameter—the difference in the heat capacity between the native and unfolded states—was assumed the same for all variants and found to be 1.8 ± 0.3 kcal mol^−1^ K^−1^. T_m_ is the temperature at which 50% of the molecules are unfolded $(\Delta G{^\circ }_{{\rm{N}}\rightleftharpoons {\rm{U}}}^{{{\rm{T}}}_{{\rm{m}}}}=0)$.

Additionally, there is an error in Table 3, in which the value “137 ± 2x” under FXN L198C should read “137 ± 2”.

