# Supplementary material for: Global Implications of Local Unfolding Phenomena, Probed by Cysteine Reactivity in Human Frataxin
Source: Sci Rep. 2019 Feb 11;9:1731. doi: 10.1038/s41598-019-39429-2 (PMC6370780; doi:10.1038/s41598-019-39429-2)
Supplement: Supplementary file 1 — Supplementary Information [file 41598_2019_39429_MOESM1_ESM.pdf]

Supplementary Information

Global Implications of Local Unfolding Phenomena, Probed by Cysteine Reactivity in Human Frataxin

Santiago E. Faraj, Martín E. Noguera, José María Delfino and Javier Santos*

Alejandro Paladini Institute of Biological Chemistry and Chemical Physics (UBA-CONICET), Faculty of Pharmacy and Biochemistry, University of Buenos Aires, Junín 956, (C1113AAD) Buenos Aires, Argentina

***Corresponding Author:** Javier Santos. Telephone: 54 011 4964 8289, ext. 108. Fax: 54 011 4 962 5457. E-mail: javiersantosw@gmail.com

Running Title: Local Unfolding Analyzed by Cysteine Reactivity

Keywords: Frataxin, conformational stability, local stability, chemical modification, cysteine residue

Abbreviations: BME: β-mercaptoethanol; CD: circular dichroism; CTR: C-terminal region; DTNB: 5,5′-dithiobis-2-nitrobenzoic acid; DTDPy: 4,4′-ditiodipiridine; FXN: human Frataxin

Materials and Methods

Protein expression and purification

Human Frataxin cDNA was kindly provided by Hèléne Puccio from the IGBMC (Strasbourg, France). FXN90-210 was prepared as described elsewhere (1). Cys-mutants were produced with reverse primers that introduced the corresponding mutation, and the PCR product was subcloned into pET9b plasmid. All variants were expressed in *Escherichia coli* BL21(DE3) and purified as previously described (2). Mass spectroscopy was used to confirm proteins' expected masses. Given that free-thiol measurements with Ellman's reagent yielded 1.0 mole of free thiol per mole of unfolded protein, we considered the use of reducing agents during our experiments not necessary. Besides, SEC-FPLC and light scattering measurements indicated that variants behave as monomers in solution, confirming the absence of intermolecular interactions. Purity was tested by SDS-PAGE and estimated to be above 98 %.

Spectroscopic Characterization of FXN Variants

Experiments were performed using a thermostated cell holder connected to a circulating water bath set at 25 °C. Circular dichroism (CD) measurements were carried out with a Jasco J-810 spectropolarimeter. Near-UV and far-UV CD spectra were collected using cells of 1.0 and 0.1 cm path-length, respectively. At least three scans were acquired at a scan speed of 20 nm min^{-1}. Values of ellipticity (θ) were converted to molar ellipticity ([θ]). Steady-state intrinsic fluorescence measurements were performed in a Jasco FP-6500 spectrofluorometer operating in the ratio mode. A 0.3 cm-path cell was used. Intrinsic fluorescence of proteins was measured using a protein concentration of 10.0 μM. Excitation wavelength was 295 nm and emission data were collected in the range of 305−450 nm. The spectral slit-widths were set to 3 nm for both monochromators.

Equilibrium Unfolding Experiments

Isothermal unfolding experiments were carried out incubating FXN with 0−8.0 M urea in a buffer solution of 20 mM Tris-HCl, 100 mM NaCl, pH 7.0 for 3 hours at room temperature. All measurements were done at 25 °C. The process was followed by far-UV CD and tryptophan

fluorescence measurements. In order to calculate thermodynamic parameters, a two-state unfolding mechanism was assumed. Data processing was performed according to Santoro and Bolen (3).

For temperature-induced unfolding experiments transition was followed by the change in CD signal at 220 nm. Protein was used at a 7.0 µM concentration, in 20 mM sodium phosphate, 100 mM NaCl, at pH values of 6.0, 7.0, and 8.0. A 1.0 cm path length cell was used. Temperature was varied from 10 to 90 °C, at a constant rate of 1 °C min^{-1}, sampling at 1 °C intervals. To obtain thermodynamic parameters, the following two-state (N⇌U) model was fitted to the data:

$$S^{\mathrm{T}_j} = \frac{\left(\mathrm{S}_\mathrm{N}^0 + l_\mathrm{N}^\mathrm{I}\,\mathrm{T}_j\right) + \left(\mathrm{S}_\mathrm{U}^0 + l_\mathrm{U}^\mathrm{I}\,\mathrm{T}_j\right)\,\mathrm{e}^{-\Delta G^{\circ\mathrm{T}_j}_{\mathrm{N}\rightleftharpoons\mathrm{U}}/\mathrm{RT}}}{1 + \mathrm{e}^{-\Delta G^{\circ\mathrm{T}_j}_{\mathrm{N}\rightleftharpoons\mathrm{U}}/\mathrm{RT}}} \quad (S1)$$

$$\Delta G^{\circ\mathrm{T}_j}_{\mathrm{N}\rightleftharpoons\mathrm{U}} = \Delta\mathrm{C_P}\left(\mathrm{T}_j - \mathrm{T_m} - \ln\frac{\mathrm{T}_j}{\mathrm{T_m}}\right) + \Delta\mathrm{H}^{\circ\mathrm{T_m}}_{\mathrm{N}\rightleftharpoons\mathrm{U}}\left(1 - \frac{\mathrm{T}_j}{\mathrm{T_m}}\right) \quad (S2)$$

where $\mathrm{S}^{\mathrm{T}_j}$ is the observed CD signal at a given T_j temperature; S_N^0 and S_U^0 are the intrinsic CD signals for the native and unfolded states, respectively; l_N^I and l_U^I are the slopes of the pre and post transition regions, respectively; $\mathrm{T_m}$ is the melting temperature ($\Delta G^{\circ\mathrm{T_m}}_{\mathrm{N}\rightleftharpoons\mathrm{U}} = 0$), $\Delta\mathrm{C_P}$ is the change in heat capacity between the N and U states, $\Delta H^{\circ\mathrm{T_m}}_{\mathrm{N}\rightleftharpoons\mathrm{U}}$ is the change in enthalpy of the N⇌U reaction between a given T_j temperature and $\mathrm{T_m}$.

Solvent accessible surface area and pKa predictions

Mutants were generated *in silico* using the crystallographic structure of FXN (PDB ID: 1EKG) as template, and the FoldX *repair* minimization routine was applied (4). Solvent accessible surface area (SASA) of the cysteine was calculated with MOL-MOL (5), and the thiol's pK_a was computed with PROPKA3 (6) and H++ (7) servers.

Iron binding and cysteine desulfurase activity

In order to determine the stability of iron-FXN complexes in solution, FXN was incubated with $FeCl_3$, and total iron in the supernatant fraction was quantified at different times, as previously described (2). Protein in the supernatant was analyzed to evaluate protein aggregation and precipitation during the incubation with the metal ion. Iron concentration was determined using a

colorimetric method based on the coordination of Fe^{2+} by 1,10-phenanthroline (8), as described elsewhere (2).

FXN-mediated activation of desulfurase activity by the NFS1-ISD11 complex was measured using the assay described by Marelja, Stocklein, Nimtz and Leimkuhler (9), with minor modifications as described elsewhere (10). Briefly, NFS1-ISD11, FXN and ISCU were mixed and supplemented with PLP, DTT and Fe^{2+}. The reaction was initiated by adding cysteine. Following a 30-minute incubation, the reaction was stopped by the addition of N,N-dimethyl-p-phenylenediamine and $FeCl_3$. Produced sulfide was used as substrate for the production of methylene blue, which was determined measuring absorbance at 670 nm (11).

Limited Proteolysis

FXN variants (1.0 mg ml^{-1}) were incubated with 0.01 mg ml^{-1} chymotrypsin for 30 seconds at 25 °C, in 20 mM Tris-HCl, 100 mM NaCl, 1 mM EDTA, pH 7.0. The reaction was immediately stopped by an addition of 1.0 mM PMSF. Samples were stored at –20°C until their analysis by SDS–PAGE and RP–HPLC followed by MALDI-TOF or ESI–MS.

Data Analysis and Model Selection

To determine the model that best explains experimental data, we assessed a single exponential function, a function made up by an exponential plus a straight line, and a double-exponential function, which were fitted by nonlinear regression to time traces. We applied the *Akaike Information Criterion* (AIC) to choose the equation that best explains the results using a minimal number of parameters with a reasonable goodness of fit (12). AIC is defined by $AIC = N \ln(SS) + 2P$, where N is the number of data points, P is the number of parameters plus 1, and SS is the sum of the weighted square residual errors. The best fitting to data is the one which results in the lowest AIC value. In our case, we found that the best equation according to AIC is a single exponential function of time. Fitting of the proposed model to experimental data and simulation of the reaction time courses were performed using the program COPASI 4.20 (13).

Some concerns on disulfide exchange reactions

In the case of small molecules such as BME or Cys-containing peptides (pL198C, L200C and pL203C), the product of the reaction of the thiolate with DTNB or DTDPy may in turn react with another molecule (R-S$^-$) to render a dimer R-S-S-R and an extra molecule of TNB$^=$ or TPy$^-$, respectively:

DTNB

$$\text{R-S}^- + \text{DTNB}^= \longrightarrow \text{R-S-S-TNB}^- + \text{TNB}^=$$

$$\text{R-S}^- + \text{R-S-S-TNB}^- \longrightarrow \text{R-S-S-R} + \text{TNB}^=$$

DTDPy

$$\text{R-S}^- + \text{DTDPy} \longrightarrow \text{R-S-S-TPy} + \text{TPy}^-$$

$$\text{R-S}^- + \text{R-S-S-TPy} \longrightarrow \text{R-S-S-R} + \text{TPy}^-$$

Although the production of TNB$^=$ or TPy$^-$ is stoichiometric ($[\text{R-S}^-]_{t=0} = [\text{TNB}^=]_{t=\infty}$), the contribution of the second reaction over the overall reaction rate will depend on the value of its rate coefficient, relative to that of the first reaction. The model we assume does not consider the second reaction, given it can be readily neglected if the first one is substantially favored. Given that in order to attain that condition it is necessary for the probe's concentration to be in excess with respect to the free thiol, this allows as well to consider the reactions to occur in pseudo-first order, indeed simplifying experimental data analysis.

Results

Conformation, flexibility and function of FXN variants

Far- and near-UV CD spectra of FXN variants showed that both the secondary structure content and the tertiary packing are native-like (Figures S2 A and B). However, some differences were observed at 270 nm, which may be compatible with local changes in the surrounding of some aromatic residues. We have previously observed similar alterations in this region of the spectra of other FXN variants (2), possibly revealing that the structure of the surroundings of some aromatic residues is very sensible to subtle changes in compactness or internal mobility. Besides, maximum fluorescence intensity was obtained at 337 nm, indicating that emission occurs from an apolar

environment. When proteins were incubated with 8.0 M urea, maximum fluorescence intensity was obtained at 352 nm, which is compatible with complete protein unfolding (Figure S2C).

To study the effect of mutations on the global flexibility of the protein, we performed limited proteolysis experiments (Figure S3). The significant resistance to chymotrypsin of this set of mutants revealed that their rigidity is comparable with that of wild-type FXN. Remarkably, longer incubations with the protease (30 minutes) lead not to further digestion of proteins. Note that the pathogenic variants FXN90-195 and FXN L198R exhibit significant sensitivity to proteases under similar condition, clearly evident after just 30 seconds of incubation (2).

Biological activity is an excellent probe for tertiary structure. The absence of large conformational alterations upon mutation is also reflected by the fact that the capability of the variants to activate NFS1/ISD11/ISCU desulfurase protein complex remains intact (Figure S4A). All variants exhibit similar activation capability to that observed for wild-type FXN. On the other hand, differences in the iron binding activity were observed among FXN variants (Figure S4B). Interestingly, the capability of maintaining iron in a soluble form correlates very well with the global stability of the variants suggesting that some aspect coupled to this property is indeed responsible of the alteration of the fine structure/dynamics at the iron binding sites locate in the acidic ridge formed by helix α1, loop α1-β1 and strand β1.

Discussion

A comprehensive model for cysteine exposition

Let us compare $\Delta G^{\circ H_2O}_{N \rightleftharpoons I}$ values calculated for FXN L203C and FXN L198C: 8 y 6 kcal mol^{-1} respectively. Clearly, the form that prevails in equilibrium is the N state in the *open* conformation and not the I state. Using the kinetic coefficients that govern the interconversion between states, we may analyze the contribution to the global (observed) modification rate of each of the reactive states distinguished in Figure 6. To illustrate this point, in Figure S8A we show the simulation of the labeling reaction of variant FXN L198C with DTNB. The kinetic coefficients that govern the reaction N⇌I, k_{IN} and k_{NI}, were obtained from folding kinetics experiments described elsewhere (14). Given

that I⇌U behaves as a rapid-equilibrium reaction, only its equilibrium constant is available. As k_{UI} is much higher than k_{IN}, we assigned to k_{UI} a value equal to 10-fold k_{IN}, and k_{IU} was calculated from $K_{\mathrm{I}\rightleftharpoons\mathrm{U}} = \frac{k_{IU}}{k_{UI}}$. We consider the modification rate, k_{mod}, to be the same for any protein state (O, I or U), and used the value obtained for the modification of BME as a free-thiol model (Figure 4C). Given that the reaction takes place under the EX2 regime (Equation 3), as experimentally verified (Figure 4 B, C, E and F), k_{close} is much higher than k_{mod}. As only the equilibrium constant for the reaction C⇌O is obtainable with the aforementioned procedure (Table 2), we consider that the value of k_{close} is 10-fold k_{mod}. The value of k_{open} is calculated from $K_{\mathrm{C}\rightleftharpoons\mathrm{O}} = \frac{k_{open}}{k_{close}}$. The fractions of each state or conformation at the beginning of the labeling reaction were those of the equilibrium dictated by the $K_{\mathrm{C}\rightleftharpoons\mathrm{O}}$, $K_{\mathrm{N}\rightleftharpoons\mathrm{I}}$ and $K_{\mathrm{I}\rightleftharpoons\mathrm{U}}$ constants (Table S3).

In order to distinguish which species contribute the most to the experimentally observed result, simulated reactions were allowed to proceed to the labeled (L) state from (*i*) any of the reactive conformations (O, I or U), (*ii*) only from I or U, or (*iii*) just from U. According to the simulations, if the reaction is allowed to proceed from any of the reactive states, the observed modification rate is analogous to that obtained experimentally (cf. Figures 4A and S8A). If, in turn, the reaction is not allowed to proceed from O, but only from I or U, the observed rate is much slower than the experimental result. This clearly shows that the fraction of reactive protein is far higher than that of I and U states taken together. Therefore, cysteine reactivity cannot be explained by global unfolding events alone, probing the existence of another reactive conformation different from I and U (Table S3).

Supplementary Figures

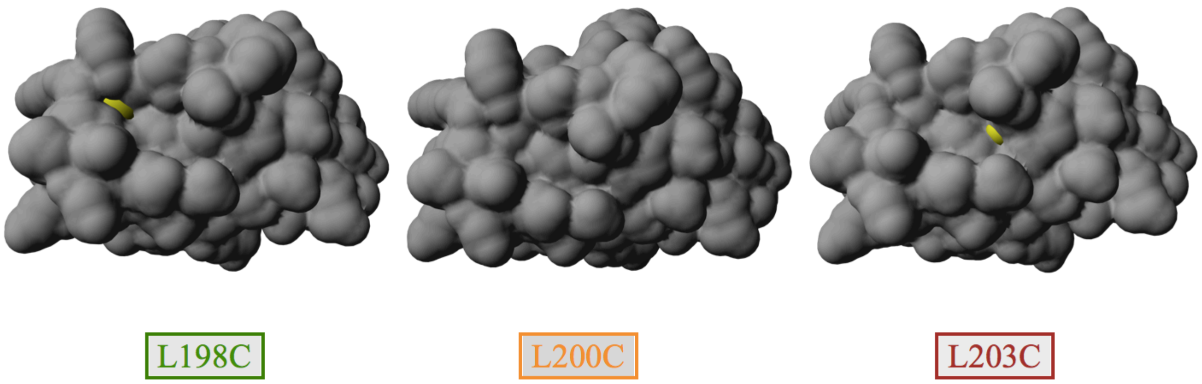

Figure S1. Solvent accessible surface area representation for each Cys variant. Structures were generated *in silico* by replacing each Leu by Cys in the crystallographic structure of wild-type FXN (PDB: 1EKG), and running the repair FoldX minimization routine over the obtained structures (RMSD: ~1.0 Å between PDB ID: 1EKG and the models). Shown views are those in which the exposition of the sulfur atom (yellow) is best noticeable. Solvent accessible surface areas (SASA) for the Cys residue of variants L198C, L200C and FXN L203C are 8.4 % (Cys), 1.3 % (Cys), 8.3 % (Cys), respectively (Table S1).

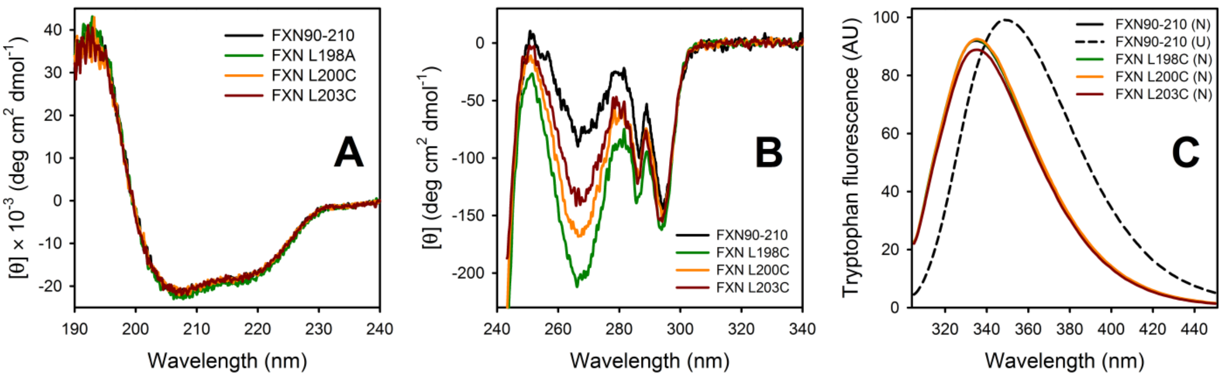

Figure S2. Spectroscopic characterization of FXN Cys-mutants. (A) Far-UV and (B) near-UV CD spectra. (C) Tryptophan fluorescence spectra (ex. 295 nm). Fluorescence spectrum corresponding to the urea-induced unfolded state (achieved by incubation with 8.0 M urea) of wild-type FXN is also shown (U).

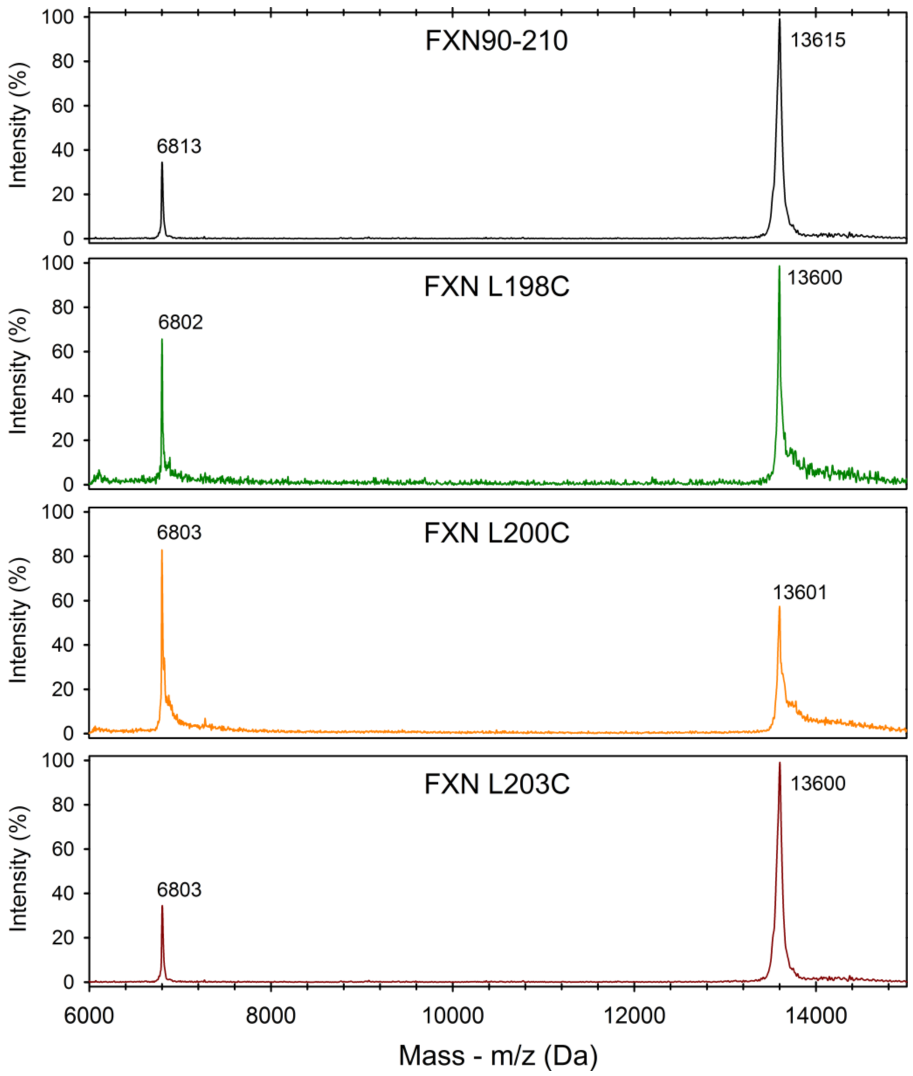

Figure S3. Limited proteolysis de FXN Cys-mutants followed by mass spectrometry. Protein samples of 1.0 mg ml^{-1} were incubated with 0.01 mg ml^{-1} chymotrypsin (1:100 protease to protein mass ratio). The reaction was stopped by the addition of 1 mM PMSF, and samples were analyzed by electrospray mass spectrometry.

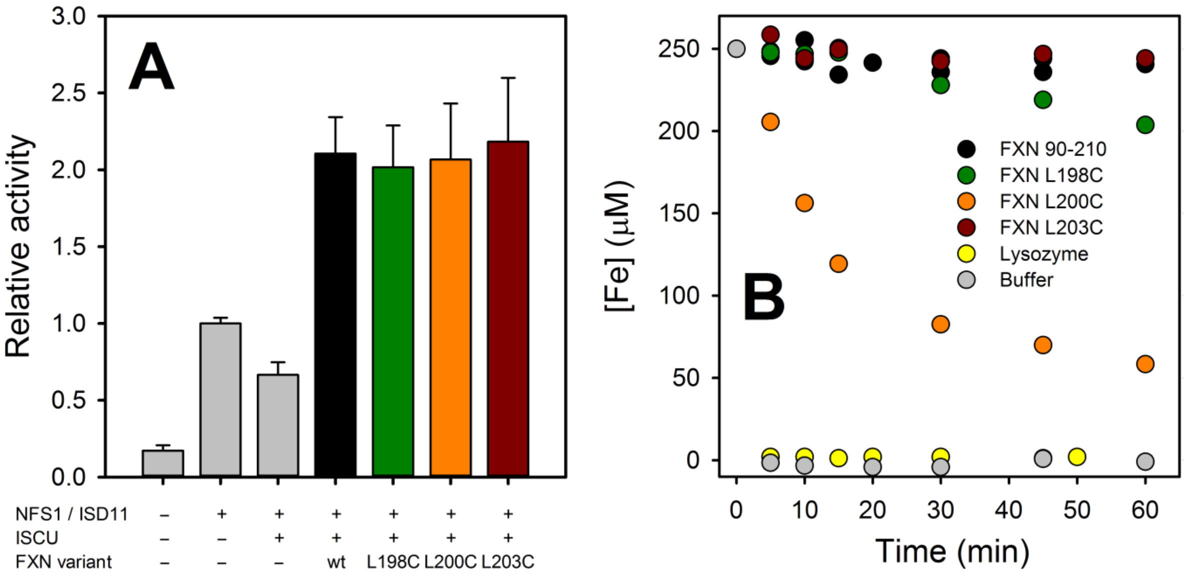

Figure S4. Functionality of FXN variants. (A) Desulfurase NFS1 activation was evaluated. Reaction controls without ISCU and/or without FXN was also included. Enzymatic and non-enzymatic (without proteins) desulfurization of cysteine to alanine and sulfide was determined by the methylene blue method. Presence or absence of each protein is indicated using + and – symbols, respectively. (B) Iron binding activity was evaluated for FXN variants. Soluble iron was measured after different incubation times in the presence of each FXN variant or in the presence of lysozyme, as a control. The reaction was performed in the absence of protein (buffer). Soluble proteins were loaded onto an SDS-PAGE before (0') or after 60-minute (60') incubation in the presence of iron to evaluate the possibility of iron-induced protein aggregation during the experiment.

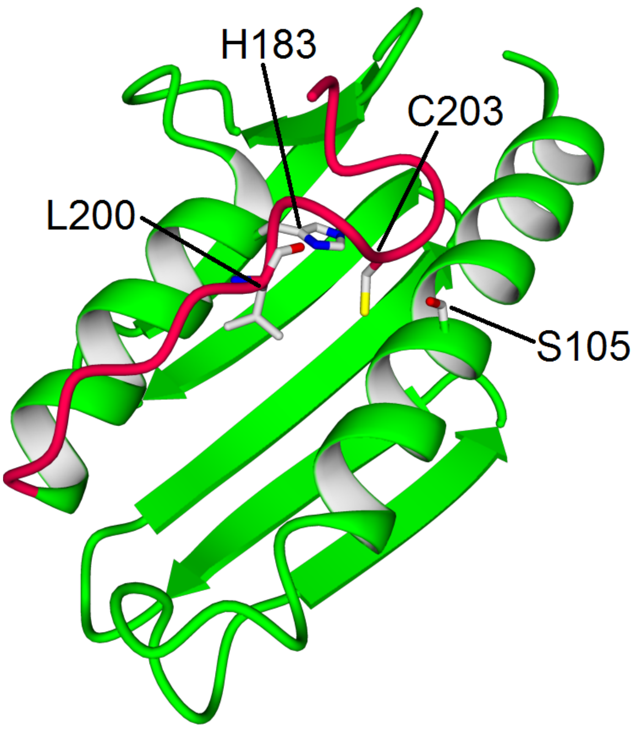

Figure S5. Ribbon representation of the structure of FXN L203C, showing the side-chains of residues S105, H183 and L200.

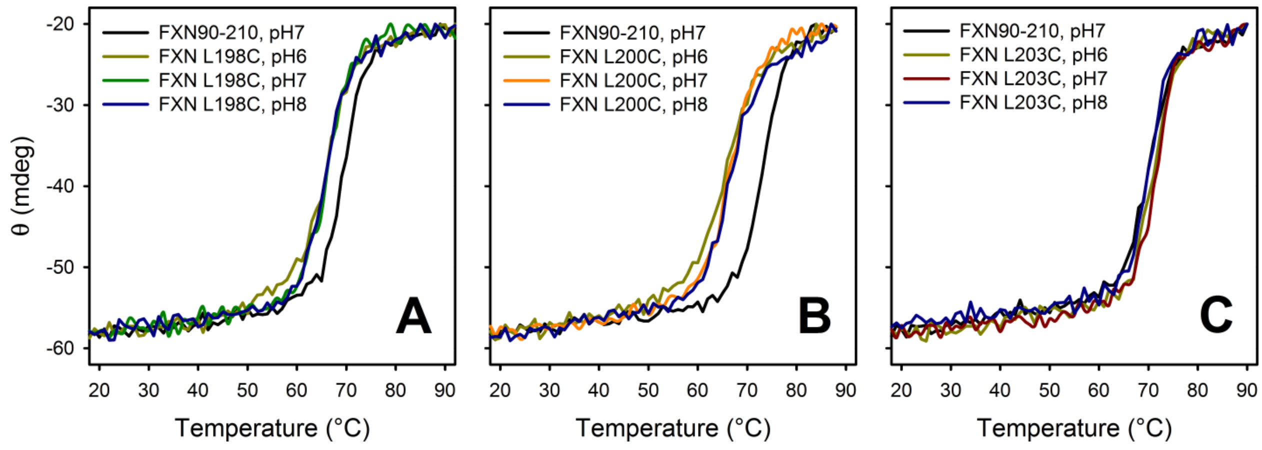

Figure S6. Thermodynamic stability dependence on pH. Thermal unfolding followed by CD at 220 nm, at pH 6.0, 7.0 and 8.0, of (A) FXN L198C, (B) FXN L200C and (C) FXN L203C.

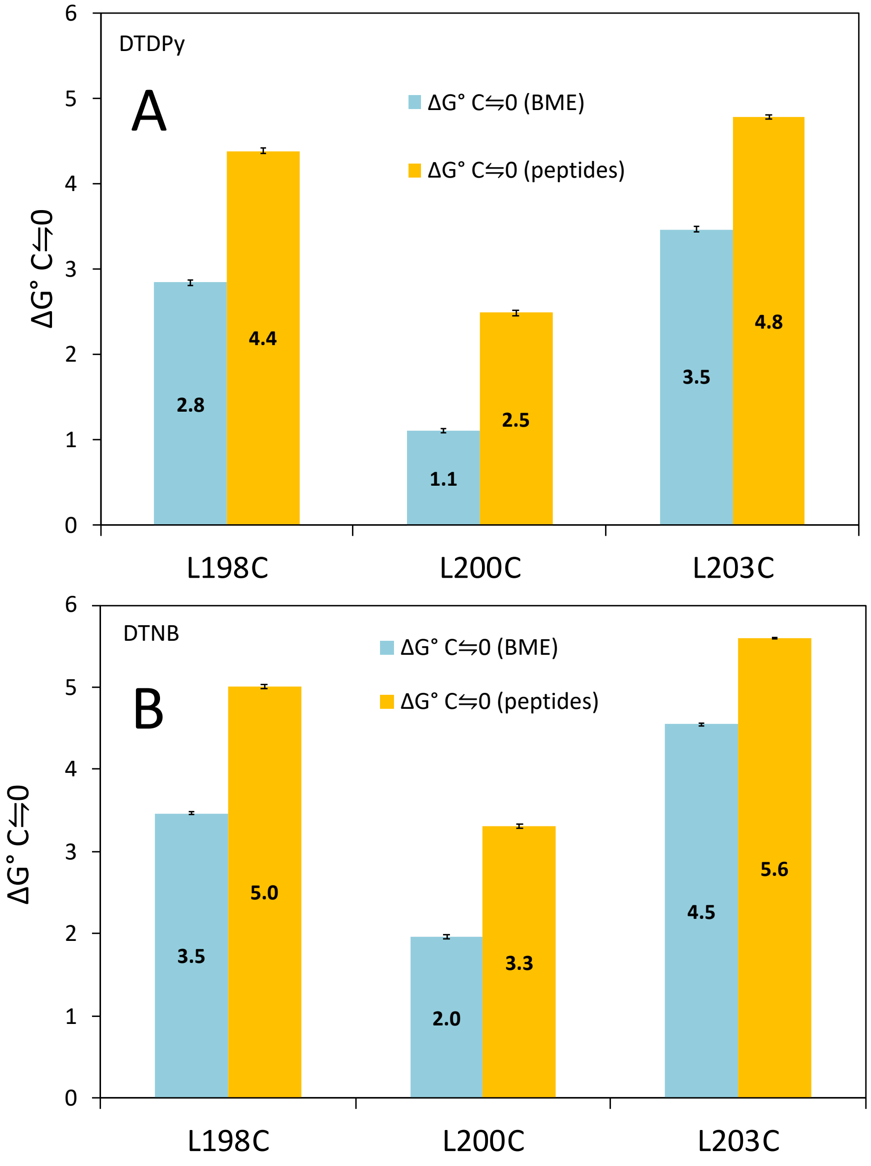

Figure S7. Local stability of FXN Cys-mutants. $\Delta G^{o}_{C\rightleftharpoons O}$ values obtained with (A) DTDPy and (B) DTNB. Error bars show ± 1 S.E of the linear regression of k_{mod} and k_{label} as a function of the probe's concentration, propagated in the free energy calculation (local unfolding, $\Delta G^{o}_{C\rightleftharpoons O}$ DTDPy and $\Delta G^{o}_{C\rightleftharpoons O}$ DTNB). The free energy differences were calculated with k_{mod} obtained by using BME as a free thiol (cyan bars) or using peptide models (orange bars, pL198C, pL200C and pL203C, respectively).

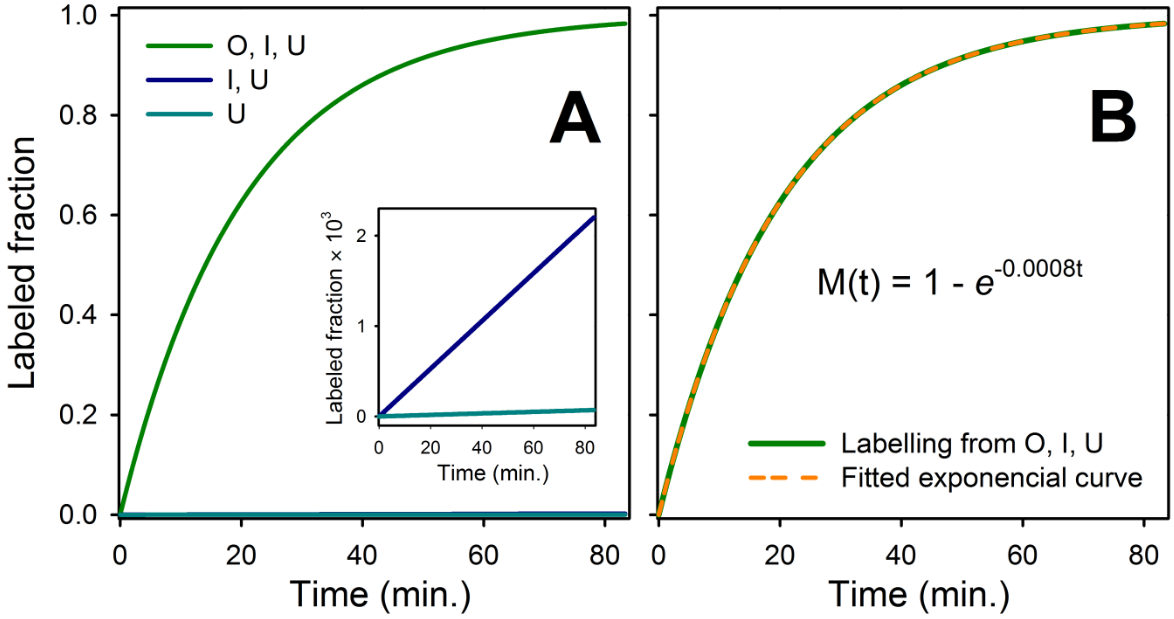

Figure S8. Simulations of the labeling reaction of mutant FXN L198C with 1 mM DTNB, using the model described in Figure 6. (A) The reaction was allowed to progress from any reactive form (O, I or U), from I or U, or from U only. The inset shows the time curse of the reaction progressing from I or U, and from U only. The kinetic coefficients were estimated as described in the main text. Used parameter values were the following: k_{close}: 3 s^{-1}, k_{open}: 0.009 s^{-1}, k_{IN}: 25 s^{-1} (14), k_{NI}: 0.001 s^{-1} ($K_{N\rightleftharpoons I}$: 4×10^{-5} (14)), k_{UI}: 250 s^{-1} ($k_{UI} = 10 \times k_{IN}$), k_{IU}: 8 s^{-1} ($K_{I\rightleftharpoons U}$: 0.032 (14)), k_{mod}: 0.3 s^{-1} mM^{-1}. (B) Best fitting of a monoexponential function of time to the simulation of the labeling reaction progressing from O, I or U (k_{obs}: 0.0008 s^{-1} mM^{-1}). Simulations were performed with COPASI (13).

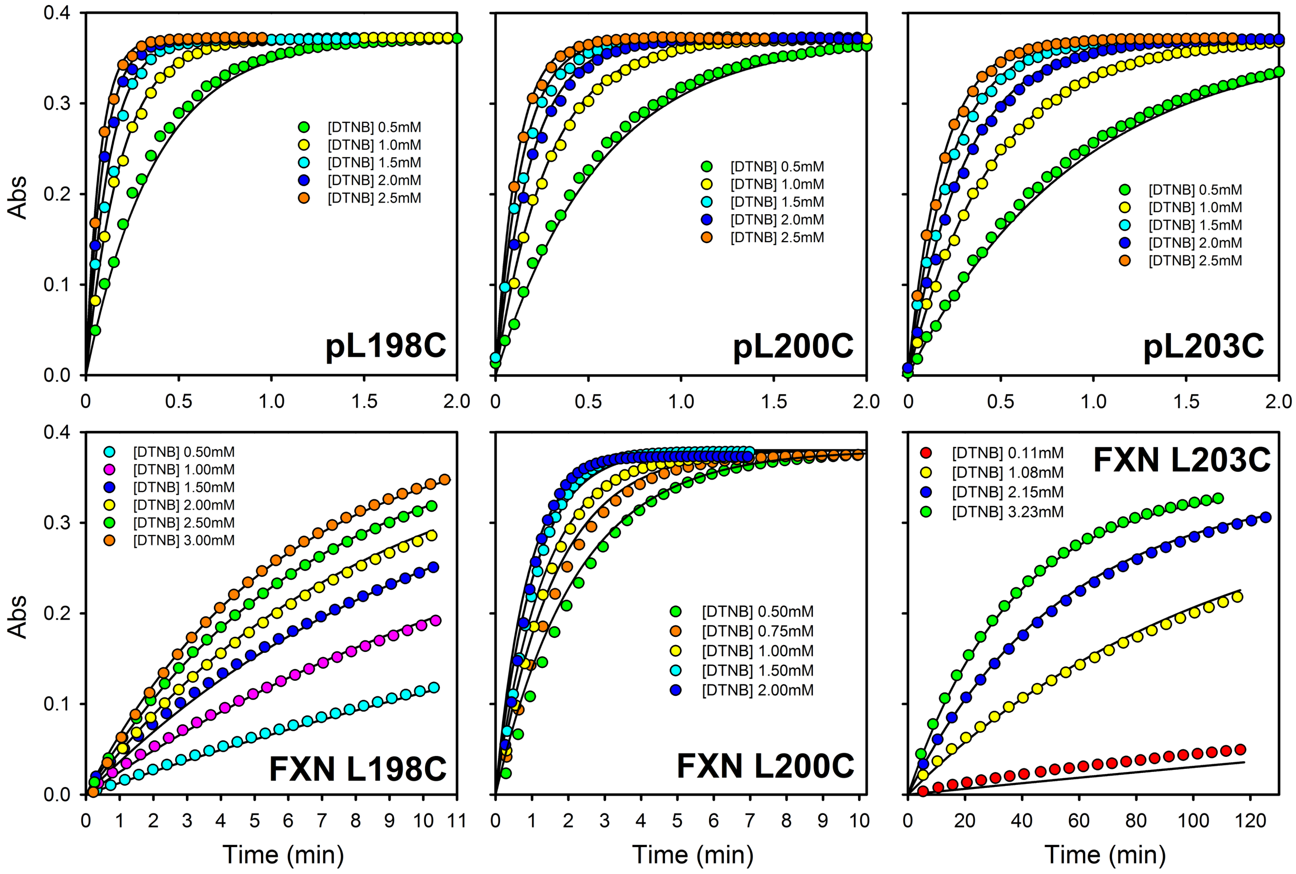

Figure S9. Adjustment of the proposed model. The model presented in Figure 6 was simultaneously fitted to experimental data obtained for FXN L198C, FXN L200C, FXN L203C and their corresponding peptides. The values of the rate constants that produce the best fitting are shown in Table 3, and were used to find the numerical solutions of the differential equations corresponding to the model (continuous lines). Used parameter values were the following: k_{IN}: 25 s^{-1}, k_{UI}: 250 s^{-1} ($k_{UI} = 10 \times k_{IN}$) and k_{IU}: 8 s^{-1} ($K_{I \rightleftharpoons U}$: 0.032); values of k_{NI} were 0.001 s^{-1} ($K_{N \rightleftharpoons I}$: 4×10^{-5}), 0.004 s^{-1} ($K_{N \rightleftharpoons I}$: 2×10^{-4}) and 0.0001 s^{-1} ($K_{N \rightleftharpoons I}$: 2×10^{-6}) for FXN L198C, FXN L200C and FXN L203C, respectively (14). Fitting of the model and simulations of curves were performed with COPASI (13).

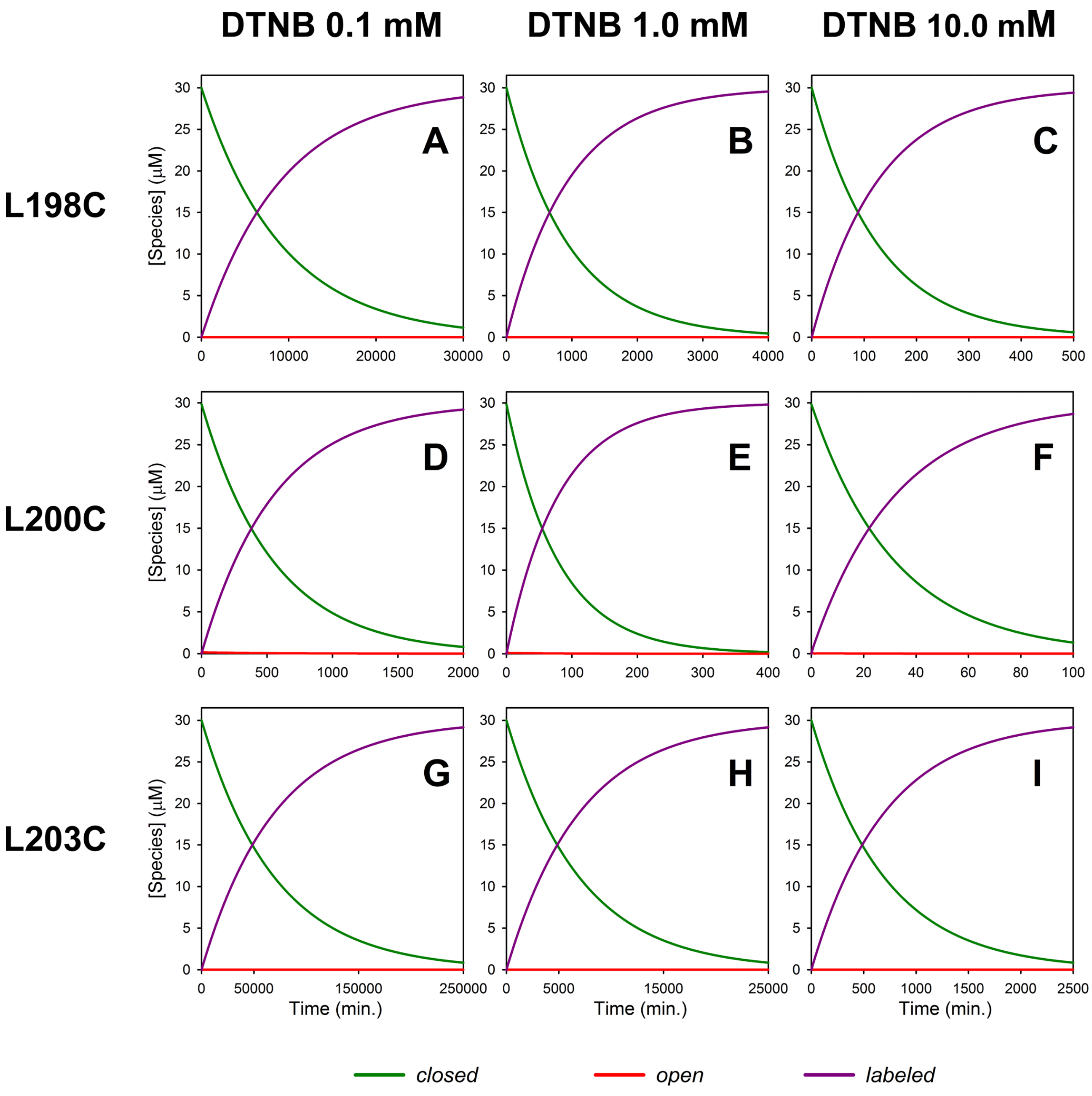

Figure S10. Simulations of the labeling reaction of L198C (A-C), L200C (D-F) and L203C (G-I) with 0.1 mM DTNB (A, D and G), 1.0 mM DTNB (B, E and H) and 5.0 mM DTNB (C, F and I), using the model described in Figure 6. Used parameter values were those from Table 3 and the following: k_{IN}: 25 s^{-1}, k_{UI}: 250 s^{-1} ($k_{UI} = 10 \times k_{IN}$) and k_{IU}: 8 s^{-1} ($K_{I \rightleftharpoons U}$: 0.032); values of k_{NI} were 0.001 s^{-1} ($K_{N \rightleftharpoons I}$: 4×10^{-5}), 0.004 s^{-1} ($K_{N \rightleftharpoons I}$: 2×10^{-4}) and 0.0001 s^{-1} ($K_{N \rightleftharpoons I}$: 2×10^{-6}) for FXN L198C, FXN L200C and FXN L203C, respectively (14). Simulations were performed with COPASI (13).

Supplementary Tables

Table S1:

TABLE S1. PREDICTIONS FOR INTRODUCED CYSTEINE RESIDUES.

Parameter	FXN L198C	FXN L200C	FXN L203C
**SASA (MOL-MOL)***	8.4 % (Cys)<br>7.2 % (S)	1.3 % (Cys)<br>0.7 % (S)	8.3 % (Cys)<br>17.7 % (S)
**pK_a (PROPKA3)**	10.2	11.5	9.7
**pK_a (H++)**	>12	>12	>12

Calculations were performed over FoldX-repaired structures.
*Solvent accessible surface area is indicated for the complete cysteine (Cys) and for the sulfur atom alone (S).

Table S2:

TABLE S2. MELTING TEMPERATURE (°C) AS A FUNCTION OF PH.

Variant	pH 6.0	pH 7.0	pH 8.0
**FXN90-210**	—	69.3 ± 0.1	—
**FXN L198C**	65.9 ± 0.2	66.4 ± 0.2	65.6 ± 0.1
**FXN L200C**	65.7 ± 0.3	66.6 ± 0.2	66.1 ± 0.2
**FXN L203C**	71.0 ± 0.2	71.3 ± 0.2	70.0 ± 0.2

†A two-state model was simultaneously fitted to the data obtained in temperature-induced unfolding experiments followed by CD at 220 nm (Figure S6). The value of the ΔC_P parameter—the difference in the heat capacity between the native and unfolded states—was assumed the same for all variants, and found to be 1.8 ± 0.3 kcal mol^{-1} K^{-1}. T_m is the temperature where 50 % of the molecules are unfolded $\left(\Delta G^{\circ T_m}_{N \rightleftharpoons U} = 0\right)$.

Table S3:

TABLE S3. RESULTS FROM 15-MINUTES SIMULATIONS OF THE LABELING REACTION OF MUTANT FXN L198C.

Specie	Initial* t=0	O / I / U† t=15 min.	I / U† t=15 min.	U† t=15 min.
**C (N)**	0.997	0.476	0.997	0.997
**O (N)**	3×10^{-3}	1×10^{-3}	3×10^{-3}	3×10^{-3}
**I**	1×10^{-6}	7×10^{-7}	1×10^{-6}	1×10^{-6}
**U**	5×10^{-8}	2×10^{-8}	5×10^{-8}	5×10^{-8}
**M**	0	0.522	4×10^{-4}	1×10^{-5}

The labeling reaction, as described by the model presented in Figure 6, was simulated for mutant FXN L198C with 1 mM DTNB, using COPASI (13). Parameters were those detailed in the caption of Figure S10.

*Initial values are those corresponding to the equilibrium distribution of species in the absence of probe.

†The reaction was allowed to progress from any reactive form (O, I or U), from I or U, or from U only.

References

1. Faraj SE, *et al*. (2013) The role of the N-terminal tail for the oligomerization, folding and stability of human frataxin. *FEBS Open Bio* 3:310-320.
2. Faraj SE, Roman EA, Aran M, Gallo M, & Santos J (2014) The alteration of the C-terminal region of human frataxin distorts its structural dynamics and function. *FEBS J* 281(15):3397-3419.
3. Santoro MM & Bolen DW (1992) A test of the linear extrapolation of unfolding free energy changes over an extended denaturant concentration range. *Biochemistry* 31(20):4901-4907.
4. Van Durme J, *et al*. (2011) A graphical interface for the FoldX forcefield. *Bioinformatics* 27(12):1711-1712.
5. Koradi R, Billeter M, & Wuthrich K (1996) MOLMOL: a program for display and analysis of macromolecular structures. *J Mol Graph* 14(1):51-55, 29-32.
6. Olsson MH, Sondergaard CR, Rostkowski M, & Jensen JH (2011) PROPKA3: Consistent Treatment of Internal and Surface Residues in Empirical pKa Predictions. *J Chem Theory Comput* 7(2):525-537.
7. Anandakrishnan R, Aguilar B, & Onufriev AV (2012) H++ 3.0: automating pK prediction and the preparation of biomolecular structures for atomistic molecular modeling and simulations. *Nucleic Acids Res* 40(Web Server issue):W537-541.
8. Marczenko Z (1976) *Spectrophotometric determination of elements* (Chalmer, R. A. Wiley, New York) pp 309-312.
9. Marelja Z, Stocklein W, Nimtz M, & Leimkuhler S (2008) A novel role for human Nfs1 in the cytoplasm: Nfs1 acts as a sulfur donor for MOCS3, a protein involved in molybdenum cofactor biosynthesis. *J Biol Chem* 283(37):25178-25185.
10. Noguera ME, *et al*. (2017) Insights on the conformational dynamics of human frataxin through modifications of loop-1. *Arch Biochem Biophys* 636:123-137.
11. Siegel LM (1965) A direct microdetermination for sulfide. *Analytical Biochemistry* 11(1):126-132.
12. Burnham KP & Anderson DR (2010) *Model selection and multimodel inference : a practical information-theoretic approach* (Springer, New York, NY [u.a.).
13. Hoops S, *et al*. (2006) COPASI--a COmplex PAthway SImulator. *Bioinformatics* 22(24):3067-3074.
14. Faraj SE, Gonzalez-Lebrero RM, Roman EA, & Santos J (2016) Human Frataxin Folds Via an Intermediate State. Role of the C-Terminal Region. *Sci Rep* 6:20782.
15. Myers JK, Pace CN, & Scholtz JM (1995) Denaturant m values and heat capacity changes: relation to changes in accessible surface areas of protein unfolding. *Protein Sci* 4(10):2138-2148.
